# Antenatal Care Interventions to Increase Contraceptive Use Following Birth in Low- and Middle-Income Countries: Systematic Review and Narrative Synthesis

**DOI:** 10.9745/GHSP-D-24-00059

**Published:** 2024-10-29

**Authors:** Ona L. McCarthy, Nasser Fardousi, Vandana Tripathi, Renae Stafford, Karen Levin, Farhad Khan, Maxine Pepper, Oona M.R. Campbell

**Affiliations:** a London School of Hygiene & Tropical Medicine, London, United Kingdom.; b EngenderHealth, MOMENTUM Safe Surgery in Family Planning and Obstetrics, Washington, DC, USA.

## Abstract

**Introduction:**

Health risks associated with short interpregnancy intervals, coupled with women’s desires to avoid pregnancy following childbirth, underscore the need for effective postpartum family planning programs. The antenatal period provides an opportunity to intervene; however, evidence is limited on the effectiveness of interventions aimed at reaching women in the antenatal period to increase voluntary postpartum family planning in low- and middle-income countries (LMICs). This systematic review aimed to identify and describe interventions in LMICs that attempted to increase postpartum contraceptive use via contacts with pregnant women in the antenatal period.

**Methods:**

Studies published from January 2012 to July 2022 were considered if they were conducted in LMICs, evaluated an intervention delivered during the antenatal period, were designed to affect postpartum contraceptive use, were experimental or quasi-experimental, and were published in French or English. The main outcome of interest was postpartum contraceptive use within 1 year after birth, defined as the use of any method of contraception at the time of data collection. We searched EMBASE, Global Health, and Medline and manually searched the reference lists from studies included in the full-text screening.

**Results:**

We double-screened 771 records and included 34 reports on 31 unique interventions in the review. Twenty-three studies were published from 2018 on, with 21 studies conducted in sub-Saharan Africa. Approximately half of the study designs (n=16) were randomized controlled trials, and half (n=15) were quasi-experimental. Interventions were heterogeneous. Among the 24 studies that reported on the main outcome of interest, 18 reported a positive intervention effect, with intervention recipients having greater contraceptive use in the first year postpartum.

**Conclusion:**

While the studies in this systematic review were heterogeneous, the findings suggest that interventions that included a multifaceted package of initiatives appeared to be most likely to have a positive effect.

## Abstract (precis)

Interventions delivered during the antenatal period that included a multifaceted package of initiatives appeared to be most likely to be effective at increasing voluntary postpartum contraception. By contrast, interventions with minimal counseling did not appear to be effective.

## Key Messages

- This systematic review adds to the limited evidence on the effectiveness of interventions delivered during the antenatal period on postpartum contraceptive use in low- and middle-income countries.
- The 31 unique interventions in 34 reports included in this review were heterogeneous, with 18 studies reporting a positive effect on contraceptive use in the first year postpartum.
- Interventions that included a multifaceted package of initiatives appeared to be more likely to have a positive effect on postpartum contraceptive use.
- Funders, policymakers, and providers may consider strengthening their support for multifaceted antenatal care interventions to promote voluntary postpartum contraceptive use to address the health risks associated with short interpregnancy intervals.

## INTRODUCTION

There is substantial evidence that short interpregnancy intervals (the time from delivery to subsequent conception is less than 18 months) increase risks of adverse maternal and perinatal outcomes such as low birth weight, small for gestational age, maternal mortality, and severe morbidity,^1^^–^^3^ and that postpartum women have strong desires to avoid pregnancy following childbirth. Yet, interest in postpartum family planning (PPFP) programs has waxed and waned since the 1960s.^4^ For example, a literature review on community-based interventions from the 1970s to 2004 aimed at improving postpartum care found a dearth of programs promoting postpartum birth spacing despite community-based FP distribution programs being widespread.^5^ However, the last decade has seen a renewed interest in voluntary PPFP, following the World Health Organization’s (WHO) issuance of guidelines in 2013 for PPFP strategies,^6^ and with strong support from the U.S. Agency for International Development and other donors through tools, such as the High Impact Practices brief for immediate PPFP,^7^ and processes to identify priority actions to support global PPFP scale-up.^8^^,^^9^

The 2013 WHO guidelines identified a continuum of 4 points of contact for PPFP, beginning with contacts during pregnancy (via facility-based ANC or community-based pregnancy-screening programs), and moving on to facility-based contacts in the intra- and immediate postpartum time periods, contacts via postpartum programs, and contacts via infant-care programs. A 2019 systematic review and meta-analysis of observational studies found that counseling during antenatal or postnatal care was a predictor of postpartum contraception use.^10^ Because antenatal care (ANC) coverage (percentage attended at least 4 visits) is 66% globally, 45% in low-income countries, 61% in lower-middle-income countries, and 91% in upper-middle- income countries^11^ and with the WHO now recommending 8 visits,^12^ there is potential ample opportunity for FP counseling during the antenatal period.

There have been several literature reviews on topics related to PPFP. A 2012 Cochrane review assessed the effectiveness of ante- and postnatal counseling on the uptake of copper intrauterine devices (IUDs).^13^ A 2010 review examined integration of FP with other health services, followed by a 2014 systematic review that also assessed the use of integration and outreach programs to promote PPFP.^14^^,^^15^ A 2015 systematic review reviewed studies of interventions explicitly intended to have an effect on the contraceptive practices of postpartum women in LMICs.^4^ A 2016 review assessed intervention strategies to decrease the unmet need for contraception among postpartum women in LMICs.^16^ Finally, a 2022 systematic review synthesized evidence on the coverage and effect of both routine FP counseling and new FP counseling interventions on postpartum modern contraceptive uptake in sub-Saharan Africa.^17^ With the exception of the most recent review, these reviews mostly included studies conducted before the 2013 WHO recommendations, and the early reviews were mostly based on studies from high-income countries.^6^^,^^12^^,^^17^ The most recent review was restricted to sub-Saharan Africa and only considered counseling interventions.^17^

This article summarizes the methods and results from a systematic review of interventions in low- and middle-income countries (LMICs) that attempted to increase voluntary postpartum contraceptive use, including the lactational amenorrhea method (LAM), through contacts with pregnant women in the antenatal period. This review aimed to describe the interventions identified and assess their effectiveness on postpartum contraceptive use within the first year postpartum and other related outcomes.

This review aimed to describe interventions that attempted to increase voluntary postpartum contraceptive use and assess their effectiveness on postpartum contraceptive use within the first year postpartum and other related outcomes.

## METHODS

### Research Questions

This review focused on any intervention (or a component of an intervention) delivered during ANC with the explicit aim of increasing voluntary postpartum contraceptive use after the index birth. Specifically, this review sought to answer the following research questions: (1) Can interventions delivered in the antenatal period increase voluntary postpartum contraceptive use? (2) What intervention components/content are present across all the effective interventions?

### Context and Population

We focused on interventions in LMICs that were delivered in the antenatal period (delivered in the community or in primary or secondary health facilities) to increase postpartum contraceptive use (including use of LAM).

### Eligibility Criteria

Studies published from January 1, 2012, to July 31, 2022, were included in this review if they were conducted in LMICs; evaluated any intervention that was delivered at some point (but not necessarily exclusively) during the antenatal period that was explicitly intended to have a distinct effect on postpartum contraceptive use (including use of LAM); were experimental (i.e., were randomized or nonrandomized trials) or quasi-experimental (e.g., controlled before-after or interrupted time-series) designs; and were full peer-reviewed articles published in French or English. Descriptive studies, qualitative research, literature reviews, opinion papers, conference proceedings, and unpublished studies were not eligible.

### Search and Screening

We used a comprehensive set of search terms around 4 themes: (1) postnatal; (2) contraceptive methods; (3) antenatal interventions, and (4) low- and middle-income countries, combined using the Boolean “AND” (the Supplement contains the full search strategy for each database). Search terms were based on those from a previous review, which included literature published through the end of 2013.^4^ We limited our search to studies published from 2012 until the end of July 2022. We also searched 2 relevant reviews published after 2012 for additional studies.^4^^,^^17^

We searched EMBASE, Global Health, and Medline and manually searched the reference lists from eligible studies included in the full-text screening for additional relevant studies. Before running the formal search, we consulted the London School of Hygiene & Tropical Medicine Library services librarian for advice. We validated our preliminary search by checking that the search results included articles of relevance suggested by experts and found in previous reviews. The search result files for each database were uploaded into a systematic review software program (Rayyan, www.rayyan.ai). After piloting the study selection process, we formally screened search results against eligibility criteria. All abstracts were double-screened, with screeners (NF, OM, OC, AC) masked to the other screeners’ decisions. Decisions were unmasked when all screening was complete. Discordant decisions were then discussed among the team and consensus was reached on whether to include or exclude these studies.

### Outcomes

The main outcome of interest was postpartum contraceptive use within 1 year of childbirth. If studies reported postpartum contraceptive use at multiple time points, these were all recorded. If a study did not report the main outcome, we required that it included a clear description of the intervention and reported at least 1 of the following outcomes: postpartum contraceptive use extending beyond a year; use of specific contraceptive methods; contraceptive continuation; postpartum unmet need; pregnancy; length of birth interval; knowledge/awareness of available methods; or intention to use a method in the postpartum period. Any other data on relevant outcomes were also extracted. All measures of intervention effect were considered (e.g., prevalence ratios or prevalence differences). Measures of intervention effect were considered significant at the 5% level.

### Data Extraction

An Excel-based extraction tool was developed by 1 team member (OM) and refined in several iterations until the final tool was agreed upon. Studies were assigned to 4 people (OM, NF, OC, and AC) for the initial data extraction. After the initial extraction was complete, the tool was separated into 4 sections: (1) general study information, (2) further details on the intervention, (3) assessment of outcomes, and (4) quality appraisal. In further discussions among the core team (OM, NF, OC), the extracted data were “harmonized” (i.e., we agreed upon a set of categories for each component of the data). For example, because the initial data extraction was based on the authors’ reports, the same study designs were often described in different ways; in these cases, we standardized the descriptions. Data were then extracted again by another team member (MP), and discrepancies were reviewed and discussed within the team.

We compiled a concise quality appraisal tool to extract data on the elements of quality most relevant to our included studies. This tool drew upon the Cochrane Effective Practice and Organization of Care risk of bias criteria and the UK National Institute for Health and Care Excellence Quality appraisal checklist for quantitative intervention studies.^18^^,^^19^ One person (OM) extracted the quality appraisal data from all studies. We used the PRISMA 2020 statement as a guide for reporting our procedures and results.^20^

### Data Synthesis

This stage aimed to understand how and why the interventions had or did not influence the different outcomes and, in doing so, to identify the factors that could explain the differences between the intervention effects. We anticipated the interventions in this review would be heterogeneous, that is, diverse regarding the features of the intervention, such as the type of intervention, its components, who delivers it, how it is delivered, when, and how often. To address this heterogeneity, we planned a narrative synthesis of the measures of intervention effect, guided by the United Kingdom Economic and Social Research Council Methods Program framework.^21^ In the preliminary synthesis, we developed an initial description of the data and organized it in terms of geographic distribution, chronological frequency of publication, and intervention type. We stratified studies by intervention type and then alphabetically under these headings. Studies were then categorized by their study design, setting, participants, intervention components (e.g., counseling, home visits, integrated, and multimodal), intervention delivery details, and contraceptive use outcomes. We looked for general patterns in effect direction and size. While we extracted data on a range of contraceptive-related outcomes, the focus of the synthesis was on postpartum contraceptive use within 1 year. Finally, we interrogated the initial patterns we observed by exploring the relationships within and between the studies. The summaries of the interventions were guided by the template for intervention description and replication checklist so that the recommended items for describing an intervention are included.^22^

## RESULTS

### Search, Screening, and Extraction

**FIGURE 1:**
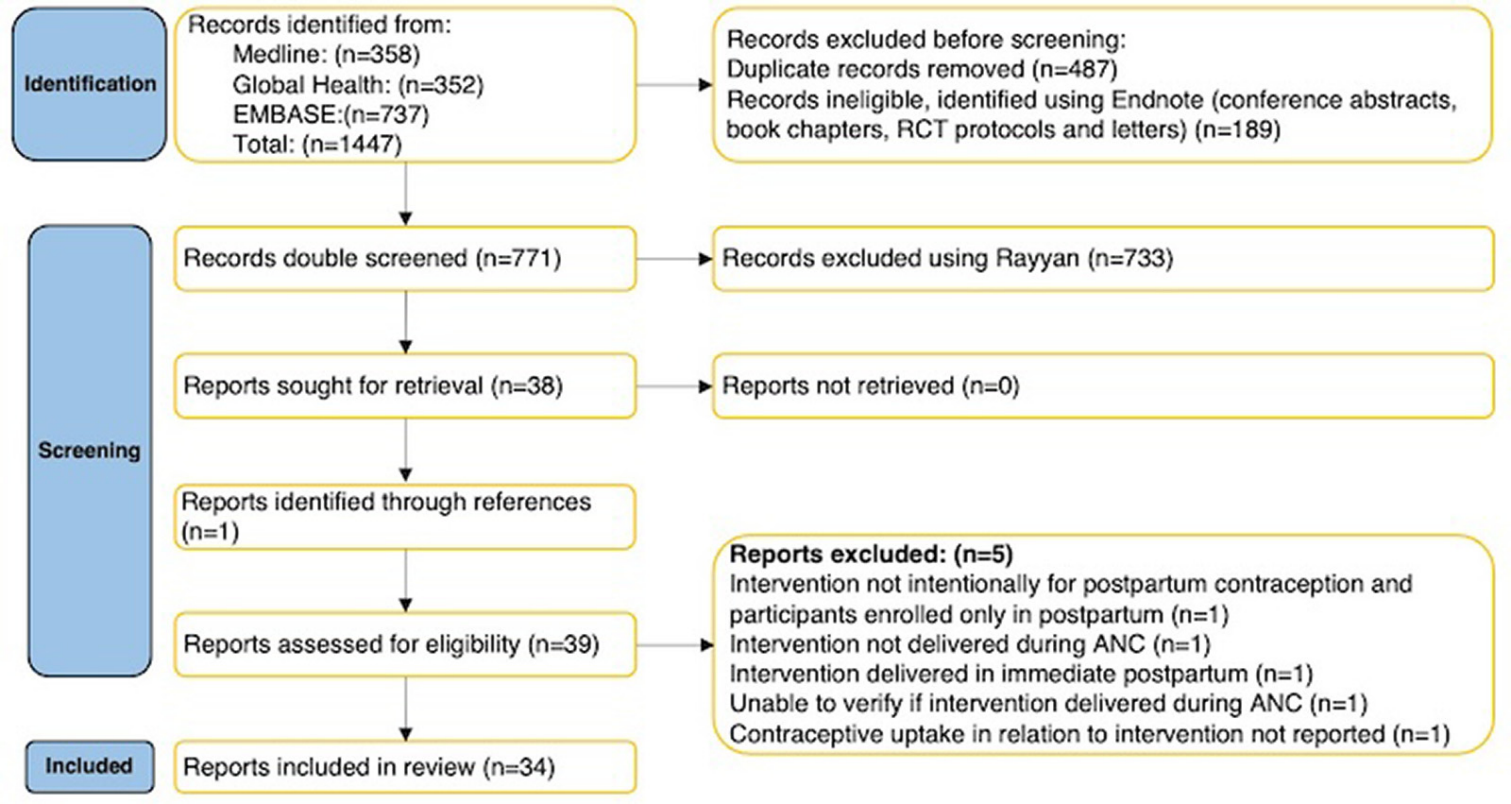
PRISMA Diagram of Included Studies Abbreviations: ANC, antenatal care; RCT, randomized controlled trial.

We began the search in July 2022 and completed double-screening in a systematic review software program on August 26, 2022. We double-screened 771 records. After unmasking the screening decisions, there were 16 discordant decisions and 30 records that required further discussion among the team. Thirty-eight records were identified for full-text review. After full-text review and double data extraction (completed March 22, 2023), 34 reports on 31 unique interventions were deemed eligible and included in the review (Figure 1).^20^^,^^23^^–^^56^

### Preliminary Synthesis

Several reports were based on the same intervention, sometimes in the same population. Although Guo et al., Huber-Krum et al., Pradhan et al., and Puri et al. had different outcomes and analytical methods on the same study in Nepal, we considered these 4 reports together and reported on the multiple outcomes under 1 study.^23^^–^^26^ Karra et al. and Pearson et al. were also based on the same intervention package as those 4 studies but were in Sri Lanka and Tanzania, respectively.^23^^–^^28^ In this case, we kept the data separate as the intervention mechanism could have been modified by the context. Similarly, Tran et al.^29^ and Tran et al.^30^ were based on the same intervention (Yam Daabo) but were conducted in Burkina Faso and the Democratic Republic of the Congo, respectively; these reports were also kept separate in the synthesis. Grouping the 4 Nepal reports together reduced the total number of studies included in the synthesis to 31.^23^^–^^56^

### Study Characteristics

**FIGURE 2:**
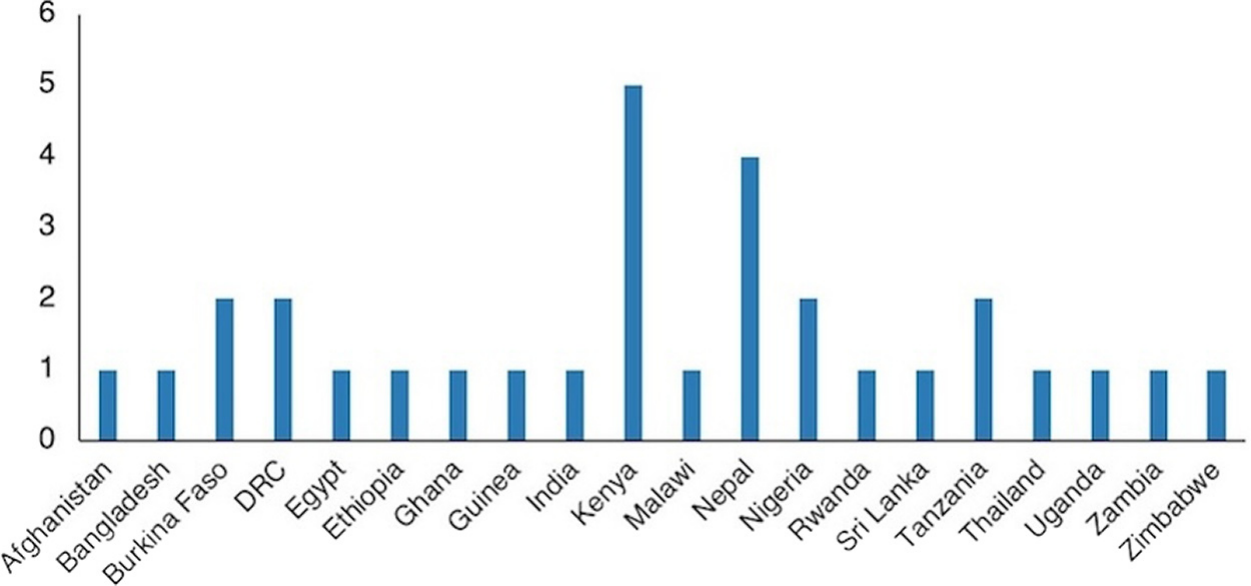
Count of Studies by Country Abbreviation: DRC, Democractic Republic of the Congo.

**FIGURE 3:**
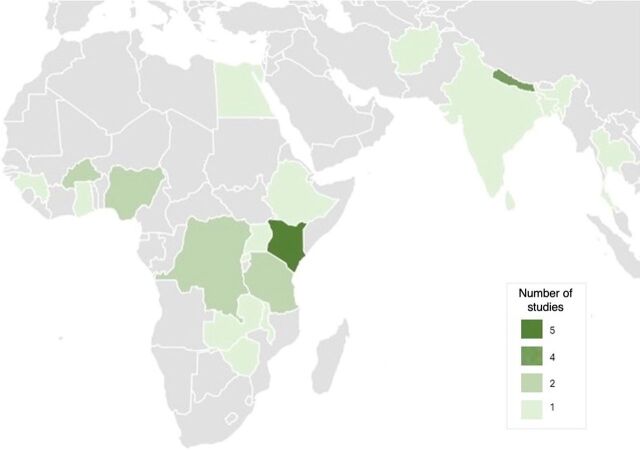
Geographical Distribution of Studies

The main characteristics of the included studies are presented in Table 1. Figure 2 and Figure 3 show where studies were conducted. The majority of studies (68%, n=21) were conducted in sub-Saharan Africa: Kenya (n=5),^31^^–^^35^ Burkina Faso (n=2),^29^^,^^36^ Democratic Republic of the Congo (DRC, n=2),^30^^,^^37^ Nigeria (n=2),^38^^,^^39^ Tanzania (n=2),^28^^,^^40^ Ethiopia (n=1),^41^ Ghana (n=1),^42^ Guinea (n=1),^43^ Malawi (n=1),^44^ Rwanda (n=1),^45^ Uganda (n=1),^46^ Zambia (n=1),^47^ and Zimbabwe (n=1).^48^ Asia had 9 reports (29%): Nepal (n=4),^23^^–26^^,^^49^^–^^51^ Afghanistan (n=1),^52^ Bangladesh (n=1),^53^ India (n=1),^54^ Sri Lanka (n=1),^27^ and Thailand (n=1).^55^ One study was conducted in Egypt.^56^

**TABLE 1.** Main Characteristics of the Included Studies, by Intervention Type

**Study**	**Country and Setting**	**Participant Characteristic at Enrollment**	**Intervention Components**	**Timing and Dose**
**Counseling interventions**				
**One-to-one**				
Ndegwa, 2014^32^	Kenya, HF: hospital	Pregnant women: 36 weeks gestation or more, attending ANC clinic at study site	ANC+: In-person intensive counseling with trained counselor was an extra effort to enhance informed decision-making.	Not specified.
Adanikin, 2013^39^	Nigeria, HF: tertiary hospital (referral center), obstetrics/gynecology department, obstetric units	Pregnant women: 28–37 weeks gestation, booked at study hospital	ANC only: In-person counseling with trained senior registrar covered information on genitalia, ovulation, fertility following birth, and modern and traditional FP methods.	3 sessions, third trimester.
Camara, 2018^43^	Guinea, lower-level HF: 5 health centers	Pregnant women: 6 months gestation or more, attending ANC visits at study health centers	ANC only: In-person counseling with trained ANC provider focused on PPFP methods (modern and traditional).	Once (15–20 minutes); during ANC visits.
Ayiasi, 2015^46^	Uganda, lower-level HF: 16 health centers	Pregnant women: 28 weeks gestation or less attending health centers for ANC	ANC only: During home visits and phone consultations, CHWs discussed risk of pregnancy soon after delivery, available options for delaying next pregnancy, and importance of regular and EBF to delay pregnancy. Women also offered phone consultations with health workers for advice.	Dose not specified; prenatal period.
**One-to-one plus pamphlet**				
Keogh, 2015^40^	Tanzania, lower-level HF: 14 antenatal clinics	Pregnant women: 3 months gestation or more	ANC only: In-person counseling with HIV post-test counselors covered benefits of spacing and limiting births; postpartum fertility and LAM; suitability of LAM based on breastfeeding plans; availability and suitability of FP methods for clients; role of condoms; referral to FP clinic and pamphlet, which covered PPFP, FP methods, and their suitability for couples living with HIV.	10 minutes of contraceptive advice after HIV post-test counseling session.
**One-to-one with spouse involvement**				
Abdulkadir, 2020^38^	Nigeria, HF: tertiary hospital, obstetrics/gynecology department, antenatal clinic	Pregnant women: 15-45 years, 32-38 weeks gestation, attending ANC at study hospital	ANC only: In-person antenatal counseling with principal author using a validated tool that includes information about the FP methods.	2 sessions; first 1 during third trimester and second 14 weeks later.
**Mixed couple and group session**				
Daniele, 2018^36^	Burkina Faso, lower-level HF: 5 (large) PHCs	Pregnant women and their male partners aged 15–45 years, 20–36 weeks gestation, attending routine check-ups at study health centers	ANC+: Private counseling sessions with auxiliary midwives or midwives covered importance of ANC and PNC, birth preparedness and signs of labor, danger signs for mother and newborn child, EBF, healthy timing and spacing of pregnancies, and PPFP. Group sessions focused on role of male partners.	3 sessions (1 hour each): group discussion between 20 weeks gestation and term, first counseling session between 20 weeks gestation and term, second session before postpartum discharge.
**Digital interventions: SMS**				
Unger, 2018^31^	Kenya, lower-level HF: government health center (MCH clinic)	Pregnant women aged 14 years or older, less than 36 weeks gestation, attending ANC at study center	ANC only: Participants classified into tracks (routine, adolescents 14–19 years, first-time mothers, women with previous cesarean delivery, and those with multiple gestations) with tailored messaging. Personalized approach that provided gestational age-appropriate educational and counseling messaging. SMS topics on ANC, FP, infant health, etc.	Weekly SMS: from enrollment to 12 weeks postpartum.
Harrington, 2019^33^	Kenya, HF: 2 public hospitals	Pregnant women and their male partners aged 14 years or older, 28 weeks gestation or more, attending ANC at study hospitals	ANC+: SMS covered general perinatal topics, and FP: available methods and their effectiveness, postpartum pregnancy risk, contraceptive safety during lactation, anticipatory guidance about side effects, community misperceptions, and dual protection.	Once a week, from enrollment (ANC visits) to 6 months postpartum.
**Educational interventions**				
**Campaign**				
Sebastian, 2012^54^	India, community: 1 district, 4 blocks, 48 villages	Pregnant women aged 15–24 years, 4–7 months gestation, max. 1 previous child	ANC only: Community workers provided counseling on healthy timing and spacing of pregnancy; postpartum care, the LAM and PPFP; educational campaign for husbands and males in community on maternity care.	During pregnancy; dose not specified.
**Group sessions**				
Maldonado, 2020^35^	Kenya, community: 4 subcounties, 77 community health units	Pregnant women: 32 weeks gestation or less, women attending ANC at a health facility	ANC+: In-person community health volunteer group educational sessions cover health and social topics relevant to antenatal, postpartum, and early childhood experiences (with an optional financial savings program).	2 60–90 minute sessions per month.
Bang, 2018^41^	Ethiopia, community: 1 district, 2 villages	Women aged 15–49 years, pregnancy status not specified	ANC+: In-person village-level sessions covered FP, safe delivery, and postpartum care. Small group classes covered FP, ANC, institutional birth, postnatal management, and neonatal/child care. One education session was given to male community leaders to encourage paternal participation in FP. Mass media was used to improve women’s awareness of maternal health. On-the-job training sessions for providers to improve their capacity in practice and provide quality of care to women. Education and mobilization of Health Development Army members to help women in their villages improve awareness of maternal health.	Interventions implemented over 2.5-year study period. 2 village-level education sessions (reaching 196 women); 39 small group classes with 3 sessions each (reaching 2,576 women).
Lori, 2018^42^	Ghana, HF: district hospital	Pregnant women aged 18 years or older, 14 weeks gestation or less	ANC only: In-person educational content and group peer support. One ANC visit dedicated to FP and EBF as a LAM.	Women encouraged to attend 7 ANC visits.
Sarnquist, 2014^48^	Zimbabwe, lower-level HF: 4 public polyclinics	Pregnant women: HIV-positive, aged 18–40 years, 26-38 weeks gestation, attending ANC at study clinics	ANC only: In-person trainers offered sessions focused on sexual negotiation skills and empowerment, information about HIV, prevention of mother-to-child HIV transmission, FP, and communication skills related to sex and FP. Various learning techniques were used, including discussions, behavior modeling, songs/ dramatizations, and role-playing.	3 90-minute group sessions; most sessions happened in antenatal period; however, 21 32% of women had at least 1 session after delivery due to late study entry or early delivery.
**Financial interventions**				
**Client vouchers**				
McConnell, 2018^34^	Kenya, lower-level HF: 2 private maternity clinics	Pregnant women aged 18-40 years, 7 months gestation or more, attending ANC at study clinics	ANC+: Vouchers given in person for free modern methods or counseling on LAM valid for 1 year and a time-limited voucher that expired 8 weeks after the estimated date of delivery. Value of voucher from US$0.92– US$6.45 depending on method; SMS reminders to use vouchers.	Vouchers given during ANC (7+ month gestation); SMS given at 5 weeks postpartum.
**Pay-for-performance**				
Engineer, 2016^52^	Afghanistan, lower-level HF: 442 facilities offering basic package of health services	Postpartum women: ever married, aged 12–49 years, up to 2 years postpartum Children: less than 5 years	ANC+: Facilities were given quarterly bonus payments based on MCH services provided: first ANC visits 1–4, skilled birth attendance cases, PNC visits 1–2, pentavalent 3 vaccination, and TB case detection. Additional annual payments also made based on 2 measures of equity of service provision, a balanced scorecard that addresses quality of services, and contraceptive prevalence rates in HF catchment areas.	Bonus amounts paid were about 6%–11% above their base salary in 2011 and increased to about 14%–28% in 2011, depending on the health worker’s cadre.
**Package of interventions**				
**Digital and one-to-one**				
Jiusitthipraphai, 2015^55^	Thailand, HF: teaching hospital	Pregnant women aged 15–19 years, gestational age not specified, women who delivered and received antenatal/postnatal care at study hospital	ANC+: In-person motivational lessons covering impacts of adolescent pregnancy, preventing subsequent pregnancies by taking oral contraceptive, mechanism of oral contraceptives, correct taking methods, forgetting to take the contraceptive, and sources of assistance. Provision of a handbook to participants. Nurses were meant to praise and encourage participants.	3 sessions: antenatal, immediate postpartum, up to 6 weeks postpartum. Weekly phone calls (5–10 minutes) for 4 weeks in postpartum period.
**Multifaceted**				
Guo, 2022^23^ Huber-Krum, 2020^24^ Pradhan, 2019^25^ Puri, 2021^26^	Nepal, HF: 6 tertiary hospitals	Postpartum women: women delivering in study hospitals (recruited after delivery and before discharge)	ANC+: FIGO’s PPIUD intervention: Training of providers (to improve counseling), information leaflet provision, establishing an information wall chart and video broadcast, training and supplies for PPIUD insertion/removal techniques, and complication management. Women received free in-person general counseling from community health volunteers on various FP methods and PPIUD-specific counseling on advantages and disadvantages, potential side effects, how to seek removal, and how long it protects from pregnancy. All counseling services, contraceptive use, and IUD removals were free.	Counseling occurred during routine ANC, at early labor, and after delivery but before discharge from hospital; provision of PPIUD in immediate postpartum and before discharge.
Karra, 2019^27^	Sri Lanka, HF: 6 tertiary hospitals	Postpartum women: women delivering in study hospitals (recruited after delivery and before discharge)	ANC only: FIGO’s PPIUD intervention: Training of providers (to improve counseling), information leaflet provision, establishing video broadcast, training and supplies for PPIUD insertion, monitoring and evaluating of counseling services.	Counseling occurred during routine ANC or after admission for delivery; provision of PPIUD in the immediate postpartum and before discharge.
Pearson, 2020^28^	Tanzania, HF: 6 tertiary hospitals	Postpartum women: 18 years or older, recruited after delivery and before discharge	ANC+: FIGO’s PPIUD intervention: Training of providers (to improve counseling), information leaflet provision, establishing video broadcast, training and supplies for PPIUD insertion, regular monitoring, and support.	Counseling occurred during routine ANC or after admission for delivery; provision of PPIUD in the immediate postpartum and before discharge.
Tran, 2019^29^	Burkina Faso, lower-level HF: 8 PHCs	Pregnant women: third trimester, attended ANC at study centers	ANC+: 3 facility-oriented interventions (i.e., refresher training of service providers, regularly scheduled and strengthened supportive supervision of providers, enhanced availability of services 7 days a week), and 3 individual-based interventions (i.e., a PPFP counseling tool, appointment cards for women, and invitation letters for partners).	Individual-based interventions delivered during third-trimester ANC visits and postnatal care follow-up visits.
Tran, 2020^30^	DRC, lower-level HF: 8 PHCs	Pregnant women: third trimester, attended ANC at study centers	ANC+: 3 facility-oriented interventions (i.e., refresher training of service providers, regularly scheduled and strengthened supportive supervision of providers, enhanced availability of services 7 days a week), and 3 individual-based interventions (i.e., a PPFP counseling tool, appointment cards for women, and invitation letters for partners).	Individual-based interventions delivered during third-trimester ANC visits and postnatal care follow-up visits.
Jarvis, 2018^37^	DRC, HF, mixed levels: 2 hospitals, 2 maternity referral centers	Pregnant and postpartum women aged 18–49 years, gestational age not specified, exiting services at study hospitals (L&D, FP, ANC, PNC, child immunization)	ANC+: In-person whole-site training for providers on quality inputs: clinical training and provision of equipment for PPIUD, training on WHO’s Medical Eligibility Criteria for Contraceptive Use, and introduction of a systematic screening and referral tool for FP (to be implemented by ANC, PNC, immunization, labor and delivery, and FP providers). Free contraceptives provided by labor and delivery and FP units.	7-day training
Karra, 2022^44^	Malawi, community: 1 city, recruited through household visits	Pregnant and postpartum women aged 18–35 years currently pregnant or up to 6m postpartum	ANC+: FP information package and private individual counseling visits: risk assessment for clinical methods and detailed information on methods switching, side effects associated with each method, benefits of contraception, birth spacing, dual protection, and male partner involvement. Financial: free transportation (taxi) service to a designated high-quality FP clinic with low waiting times; Free FP services at designated clinic or financial reimbursement for any FP services received at other clinics; and reimbursement for treatment costs if woman experienced any contraindications or side effects related to use of FP. Free phone consultations to discuss side effects if needed.	1 counseling session within 1 month after administering baseline, 5 shorter follow-up sessions spaced over 2 years; sessions lasted up to 1 hour.
Espey, 2021^45^	Rwanda, HF, mixed levels: 2 high-volume hospitals, 4 health centers	Pregnant and postpartum women (up to 6 weeks postpartum)	ANC+: Group and individual counseling to expectant mothers (with possibility of partner involvement) on PPFP, integration of FP counseling in ANC, labor and delivery, and infant vaccination services. Provider training on PPIUD insertion/removal. Higher provider reimbursement for IUDs compared to implants. Engagement with Ministry of Health stakeholder.	Antenatal period up to 6 weeks postpartum. Group sessions and individual counseling lasted 20 minutes each.
Wu, 2020^51^	Nepal, community: 1 rural municipality	Postpartum women: married, aged 15–49 years, up to 1 year postpartum	ANC+: In-person home-based ANC and PNC counseling by CHW on clinical topics included recommendations and reasons for birth spacing, contraceptive efficacy, contraindications, timing for initiation of PPFP, and facilities where each method; training materials emphasized best practices for contraceptive counseling, such as shared decision-making, respect for patient autonomy, and anticipatory guidance on potential side effects, and home-based childcare and counseling.	General contraceptive counseling occurred at the eighth month ANC home visit, and patient-centered contraceptive counseling was offered at PNC months 1, 5, and 10.
Ahmed, 2015^53^	Bangladesh, community: 4 rural unions	Pregnant women: gestational age not specified (recruitment must have happened <32 weeks)	ANC+: CHWs discussed the importance of pregnancy spacing, effective LAM use, and LAM transition (+ flyers summarizing information). In addition, CHWs provided oral contraceptives, condoms, and injectables. Community-based monthly meetings to discuss importance of pregnancy spacing and PPFP, including LAM.	Household visits every 2 months (antenatal and postnatal period; pregnancy-surveillance visits) and community-based meetings every month.
Cooper, 2016^56^	Egypt, community: households, 6 governorates	Postpartum women: women with a child aged 24 months or younger	ANC only: Home visits and community-based group discussion counseling covered benefits of FP, healthy timing and spacing of pregnancies, postpartum return to fecundity, and risk of pregnancy after childbirth, LAM, and gender roles. Mobile clinics offered free medical care, including FP. Cooperation with health directors and pharmacists to improve access to FP methods.	During pregnancy and up to 24 months postpartum
**Systems strengthening**				
Buser, 2021^47^	Zambia, lower-level HF: 40 HFs	Postpartum women aged 15 years or older, women who gave birth in one of the study facilities in the previous 13m	ANC+: Improving MWHs through infrastructure, equipment, and supplies to address the need for higher quality, safer MWHs; policies, management, and financial structures; and linkages to health systems with skilled midwives (incl. participation of women living in MWH in maternal and child education courses at HF)	Around births (women in MWH could attend ANC and PNC)
Maru, 2017^49^	Nepal, mixed HF and community: hospital and community (CHWs in 14 community clusters)	Pregnant women aged 15–49 years, gestational age not specified	Unclear: Evaluated improvements to existing public-private partnership program: strengthening CHW active surveillance, integrating digital health information, and increasing monitoring and supervision capabilities. CHWs continuously survey population for new pregnancies, assist in attaining laboratory and ultrasound testing to identify high-risk pregnancies, and follow those pregnancies through postpartum period. Patient data are collected in an open-source electronic health records platform, and key performance measures are tracked and incorporated into the financial contract.	Not specified
**Training intervention**				
**Providers**				
Dhital, 2021^50^	Nepal, Mixed, HF and community: 2 major referral hospitals and catchment area of 23 peripheral HFs	Female community health volunteers and postpartum women	ANC only: Training for providers covered different PPFP methods and advantages and disadvantages of each and PPIUD in more detail as it was only long-acting reversible method available in immediate postpartum period in Nepal.	Not specified

Abbreviations: ANC, antenatal care; ANC+, antenatal period and other periods; CHW, community health worker; DRC, Democratic Republic of the Congo; EBF, exclusive breastfeeding; FIGO, Federation of International Gynaecology and Obstetrics; FP, family planning; HF, health facility; IUD, intrauterine device; LAM, lactational amenorrhea method; MCH, maternal and child health; MWH, maternity waiting home; PHC, primary health center; PNC, postnatal care; PPFP, postpartum family planning; PPIUD, postpartum intrauterine device; SMS, short messages service; WHO, World Health Organization.

**FIGURE 4:**
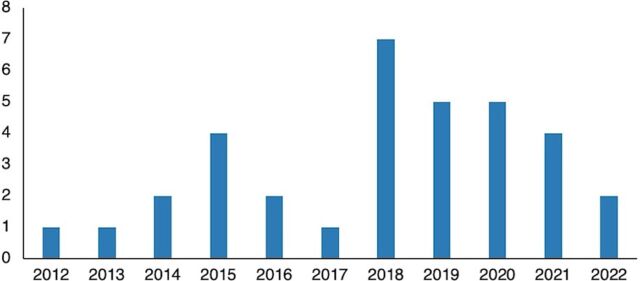
Publication Year of Included Studies

Reports were published in all years from 2012 to 2022 inclusive, with 68% (23 of 34) published in the previous 5 years (2018–2022) (Figure 4, n=34, the grouped Nepal reports are separated in this figure).

Around half of the study designs (52%, n=16) were randomized controlled trials, and half (48%, n=15) were quasi-experimental. Most studies (77%, n=24) were conducted in health facility settings: hospitals (n=9), lower-level facilities (n=11), mixed-level facilities (n=2), and mixed health facilities and community (n=2). The remaining 7 studies (23%) were conducted in community settings only.

Just over half the studies specified that participants were pregnant women (55%, n=17). The rest of the studies enrolled postpartum women only (23%, n=7), pregnant and postpartum women (10%, n=3), pregnant women and their male partners (6%, n=2), women aged 15-49 (pregnancy status not specified) (3%, n=1), and postpartum women and female community health volunteers (3%, n=1). When women were enrolled in the postpartum period, they were still assessed for the effect of an intervention delivered in the antenatal period.

#### Interventions

Details of the intervention components are presented in Table 1. Twelve studies described interventions in the antenatal period only and 18 in the antenatal and other periods, including in the intra- and postpartum periods. One was unclear. Despite the interventions being heterogeneous, we were able to categorize them into 6 main types: counseling (23%, n=7), digital (6%, n=2), educational (16%, n=5), financial (6%, n=2), package (45%, n=14), and training of providers (without an explicit follow-up intervention with pregnant women) (3%, n=1). Interventions were classified as package if they contained at least 2 distinct components. Interventions were classified as counseling if the provision of counseling was the dominant component (e.g., 1 intervention provided one-to-one counseling but also offered a pamphlet containing similar information and was classified as counseling rather than package) and if they were delivered one-to-one or to a couple, whereas interventions delivered to a group only were classified as educational. The distinction between counseling and education was not always clear (e.g., Bang et al. described an educational intervention that consisted of using village-level sessions and small-group sessions; we could not be sure what the village-level sessions comprised [i.e., they could have consisted of one-to-one or couple sessions]).^41^ In unclear cases, we classified the intervention according to the description used by the authors. We further categorized the types into 13 subtypes: counseling: one-to-one (13%, n=4); counseling: one-to-one plus pamphlet (3%, n=1); counseling: one-to-one with spouse involvement (3%, n=1); counseling: mixed couple and group sessions (3%, n=1); digital: SMS (6%, n=2); educational: campaign (3%, n=1); educational: group sessions (13%, n=4); financial: client vouchers (3%, n=1); financial: provider pay-for-performance (3%, n=1); package: multifaceted (35%, n=11); package: digital and one-to-one education and counseling (3%, n=1); package: systems strengthening (6%, n=2); and training: providers (3%, n=1).

Although the interventions were heterogeneous, they could be categorized into counseling, digital, educational, financial, package, and training of providers.

Most counseling interventions consisted of multiple sessions during pregnancy. One^36^ consisted of mixed sessions with 2 couple counseling sessions and 1 male group session. One intervention was a small subcomponent of a post-HIV test counseling that included sexual negotiation skills and empowerment, information about HIV, prevention of mother-to-child transmission, FP, and communication skills related to sex and condom use.^40^ Half the counseling interventions (n=4) were delivered by providers, mostly in ANC clinics.

The 2 digital interventions were delivered by SMS. Both contained a 2-way element, and the trial^31^ also included a 1-way SMS arm and classified participants into tracks (routine, adolescents 14–19 years, first-time mothers, women with a previous cesarean delivery, and those with multiple gestations), with tailored messages for each.

The educational campaign was led by community health workers.^54^ Of the 4 educational group sessions, 1 was delivered by study trainers^48^ and 1 by community health volunteers^35^; Lori et al.^42^ and Bang et al.^41^ did not mention who delivered the educational sessions.

One financial intervention targeted clients,^34^ included pregnant women attending ANC at a private-sector health care facility, and provided vouchers for free contraceptive methods. The vouchers had different validity periods; 1 was valid for 1 year and 1 valid for 8 weeks after the estimated date of delivery. Participants were also randomly assigned to receive an SMS reminder to use the vouchers, which was sent at 5 weeks postpartum. The other financial intervention was provider-targeted and used “pay for performance,” where facilities were given bonus payments based on the number of maternal and child health services that they provided (ANC, skilled birth attendance, postnatal care, pentavalent vaccination, and TB case detection), with additional annual payments based on a balanced scorecard that addressed quality of services, and the contraceptive prevalence rates in the health facility catchment areas.^52^

One of the 2 package interventions that focused on system-strengthening was based on accountable care principles,^49^ where “a group of providers were held accountable for achieving pre-specified outcomes for a specific population over a period of time for an agreed cost.” This involved strengthening community health worker active surveillance, integrating digital health information, and increasing monitoring and supervision capabilities. The other system-strengthening package intervention had multiple interacting components, including improvements in maternity waiting home infrastructure (in the form of equipment and supplies), policies, management and financial structures, and linkages to health systems with skilled midwives.^47^ Three package interventions consisted of one-to-one counseling plus other components. One study^53^ added community-based meetings led by community mobilizers, flyers, and distributed contraceptives. The second^45^ added provider training on insertion and removal of postpartum intrauterine device (PPIUD) and a refresher on postpartum implants, financial incentives, and stakeholder engagement. The third study^55^ included a digital component using computerized media to promote self-efficacy in oral contraceptive use. The remaining multifaceted package interventions contained a wide variety of components, which included counseling, training of providers, and the provision of equipment.

The training intervention consisted of training providers on PPFP, including the advantages and disadvantages of each method, focusing on immediate PPIUD.^50^

### Characterizing the Included Studies

#### Outcomes

Twenty-four of the 31 studies reported on the main outcome of interest: postpartum contraceptive use within 1 year after birth. Eight studies reported prevalence at 12 months exactly. Others reported use at 48 hours (about 2 days), 1 week, and various other time points and periods within 12 months, with 1 study reporting the primary outcome up to 24 months. The other 7 included studies reported on 1 of the additional outcomes.

We assessed the studies regarding the main outcome of interest (postpartum contraceptive use within 1 year after birth) by considering the intervention components and effect sizes within the studies that demonstrated a positive effect against those that did not find a positive intervention effect. The outcome measures were heterogeneous (Table 2), which supports our decision to conduct a narrative synthesis rather than a meta-analysis.

**TABLE 2.** Summary of Main Effect Sizes, by Outcome Type^a^

**Study**	**Postpartum Contraceptive Use Within 1 Year of Birth**	**Use of Specific Methods of Contraception**	**Other Outcomes**
**Counseling interventions**			
Ndegwa, 2014^32^		Post-placental IUD insertion: 63.3% intensive vs. 64.3% routine *P*=.23	
Adanikin, 2013^39^	6 months: intervention group reported higher modern contraceptive use (57.4% vs. 35.4%; *P*=.002) and less use of traditional methods (19.8% vs. 32.3%; P=.044)	Precise method used postpartum (*P=.*061):Condom: 30.7% vs. 18.2%IUD: 12.9% vs. 11.1%POP/COC: 6.9% vs. 4.0%Injectables: 5.0% vs. 2.0%Implants: 0 vs. 0Sterilization: 2.0% vs. 0LAM: 13.9% vs. 21.2%Calendar: 4.0% vs. 2.0%Withdrawal: 2.0% vs. 9.1%	
Camara, 2018^43^	6 months: no difference in use of any FP method (4.8% vs. 5.7 in intervention; *P*=.708);No difference in use of modern FP method (3.2% vs. 4.6% in intervention; *P*=.473)9 months: no difference in use of any FP method (2.7% vs. 6.7% in intervention; *P*=.064);Higher uptake of modern FP methods in intervention group (1.1% vs. 5.7% in intervention; *P*=.024)	6 months: no difference in choice of FP method (*P*=.282): condoms (2.1% vs. 2.1%), pills (0.0% vs. 2.1%), IUD (0.0% vs. 0.0%), injectable (0.0% vs. 0.0%), traditional methods (1.6% vs. 1.0%)At 9 months: no difference in choice of FP method *(P*=.058): pills (0.0% vs. 0.5%), injectable (0.5% vs. 5.2%), implant (0.5% vs. 0.0%), traditional methods (1.6% vs. 1.0%).The authors intended to group LAM with modern methods but could not verify its accurate measurement.	At 9 months, women cited more FP methods in intervention group.More women with postpartum FP intention in the intervention group at 6 months (88% vs. 69%, *P*<.01), as well as at 9 m months (78% vs. 54%, *P*<.001). However, these proportions were similar at time of inclusion just after counseling session.Also asked for reasons for not using FP methods; common ones: preference to abstain from sexual intercourse till child walks, unavailability of desired FP method, husband does not want it.
Ayiasi, 2015^46^	12 months: Only 28.2% (control) and 31.6% (intervention) of mothers were current users of modern contraceptives. Although there was slightly higher proportion of current users in the intervention arm, this difference was not statistically significant (aRR: 1.10; 95% CI=0.51, 1.82; *P*=.810).		About half of postpartum women, 47.1% (control) and 49% (intervention) arm had considered delaying the next pregnancy among the current noncontraceptive users, signifying unmet needs for contraceptive use. Of these, 71.4% in control and 87% in intervention had considered using a modern FP method. In preliminary analysis, risk of being willing to use was 1.5 times higher among intervention group, but this difference was not statistically significant after adjustment (aRR: 0.98; 95% CI=0.53, 1.82; *P*=.955).Pregnancy: Intervention arm (3.3% vs. 5.7%; *P*=.302)No difference in breastfeeding practices.
Keogh, 2015^40^	At 6–15 months (median 10.5 months): No evidence of an association between antenatal counseling and starting FP		At 6–15 months (median 10.5 months):No evidence of an association between antenatal counseling and stopping FP, unmet need, and repeat pregnancy.
Abdulkadir, 2020^38^	12–20 weeks (2.8–4.6 months): intervention group reported higher contraceptive use (48.5% vs. 31.0%, *P*=.0001 based on Mc Nemar’s X2)		Significant predictors of uptake: occupation, education, husbands’ participation
Daniele, 2018^36^	3 months: Positive effect on use of any contraceptive method (57.0% vs. 49.3% in control, RD=7.7 [1.2 to 13.6], RR=1.16 95% CI=1.04, 1.30)8 months: Positive effect on use of any contraceptive method (70.6% vs. 64.4% in control, RD=6.5 95% CI=1.0, 12.1; RR=1.10 95% CI=1.02, 1.20)Positive effect on use of effective modern contraceptive methods (59.6% vs. 53.1% in control, RD=6.4, RR=1.12 95% CI=1.01, 1.24).	8 months: positive effect on use of long-acting or permanent contraception (30.7% vs. 22.9% in control, RD=8.1, RR=1.33 95% CI=1.09, 1.62)	Intervention was associated with reduction of unmet need for contraception 8 months postpartum (14.2% vs. 18.7% in control, RD= −4.8, RR=0.75; 95% CI=0.57, 0.98Also looked at timely initiation of effective modern contraception, Unmet need for contraception 8 months postpartum.
**Digital interventions**			
Unger, 2018^31^	16 weeks (3.7 months): Contraceptive use was significantly higher in both intervention arms (1-way SMS: 72% and 2-way SMS: 73%; *P*=.03 and 0.02 versus 57% control, respectively). However, this difference was not significant when correcting for multiple comparisons.At 10 and 24 weeks (2.3 months and 5.5 months): No difference in contraceptive uptake between groups.	LARCs use similar across arms:One-way versus control, RR 1.16, 95% CI=0.44, 3.03; *P*=0.772-way versus control, RR 1.41 95% CI=0.57, 3.51; *P*=0.46) with only 25 (11%) of all contraceptive users using long-acting, reversible contraception methods (intrauterine devices and implants), the majority implants.Women in both intervention arms were significantly more likely to EBF at 10 weeks and 16 weeks than women in the control arm. The probability of EBF to 24 weeks postpartum was higher in both intervention groups than in the control, but only statistically significant in the 2-way messaging group [0.49 in 1-way, 0.62 in 2-way, and 0.41 in control, (*P*=.30 and .005 for 1-way and 2-way vs. control, respectively)]	Contraceptive continuation high among women starting contraception at 10 weeks; however, 44 (30%) of contraceptive users across all arms switched methods between 10 and 24 weeks.
Harrington, 2019^33^	6 months: use of any contraceptive method higher among women in the SMS group (aRR=1.19; 95% CI=1.01, 1.41)	6 months: use of highly effective methods higher among women in the SMS group (aRR=1.26; 95% CI=1.04, 1.52). No difference observed in use of LARC/permanent contraception (aRR=0.96; 95% CI=0.91, 1.02).At 6 months, 31.7% of all attendees were using injection. Implant users made up 25.4% of participants at 6 months. No participants reported LAM as their method of contraception at the 6 months visit.	Contraceptive discontinuation at 6 months was comparable in the SMS and control groups at 1.6% (*P*=.96).
**Educational interventions**			
Sebastian, 2012^54^	9 months: higher proportion of women in the intervention group than of those in the comparison group reported modern contraceptive use (57.0% vs. 30.1%, *P*≤.01)	9 months – choice of methods:Pill: 13.8% (intervention) vs. 7.1% (control)Condoms: 40.9% (intervention) vs. 22.6% (control)IUD: 1.9% (intervention) vs. 0.2% (control)Sterilization: 0.4% (intervention) vs. 0.2% (control)Traditional method: 18.9% (intervention) vs. 25.3 (control); *P*≤.014 months – LAM:23% (intervention) vs. 13% (control)	Knowledge of the various contraceptive methods (including LAM) was significantly higher in the intervention group compared with the comparison group at 4 months postpartum; these differences were even greater at the 9-month postpartum survey.
Maldonado, 2020^35^	12 months: increased contraceptive adoption in intervention clusters (RD 7.2%, 95% CI=2.6, 12.9, *P*=.034)	12 months: increased EBF in intervention clusters (11.9% 95% CI=7.2%, 16.9%; *P*=.14).No statistically significant effect on adoption of LARCs (RD=7.1% 95% CI=0.9%, 13.3%; *P*=.099).	
Bang, 2018^41^	18-19 months after the baseline survey: In intervention group, contraceptive prevalence increased from 31.3% to 61.8% (in comparison group: from 33% to 35.5%) (*P*=.065)		The intervention group showed significantly greater increases in knowledge about FP compared to the comparison group (*P*<.038).
Lori, 2018^42^	12 months: Women who participated in group ANC had higher odds of using a modern or non-modern method of contraception (aOR= 6.690, 95% CI=2.724, 16.420)	12 months: Women who participated in group ANC had higher odds of using a modern FP method than those in individual care (aOR=8.063, 95% CI=2.887, 22.524).Women enrolled in group ANC had nearly three-fold odds of EBF for more than 6 months compared with women in individual care (aOR=2.84, 95% CI= 1.298, 6.216).	Women who participated in group ANC were more likely to demonstrate intention to use FP immediately postpartum than those who were in individual care (63.0% vs. 31.6%, X^2^=16.49, *P*<.001)
Sarnquist, 2014^48^	3 months: uptake of LARCs in intervention (87.1%) and standard of care (81.8%) group (*P*=.34). Uptake of other modern FP methods in intervention (9.7%) and standard of care (9.1%) group (*P*=.12).	Use at 3 months PP (Intervention v control)IUD: 1.6% v 9.1%, *P*=.12Implant: 85.5% v 72.7%, *P*=.11	Identified IUD as effective at preventing pregnancy, 3 months PP (Intervention vs. control)85.5% v 56.3%, .002
**Financial interventions**			
McConnell, 2018^34^	22 weeks (after estimated date of delivery; 5.1 months): increased probability of using modern contraception among those with standard voucher + SMS (RD=25% [6%, 44%]). None of the other treatment arms were estimated to statistically significantly increase the likelihood of modern contraceptive use	22 weeks (after estimated date of delivery): increased probability of using LARCs among those with standard voucher + SMS (RD=20% [0%, 41%]). None of the other treatment arms were estimated to statistically significantly increase the likelihood of LARC use.	
Engineer, 2016^52^			23-25 months after P4P rollout- current use of modern FP methods: 10.7% vs 11.2% (*P*-value: 0.90)
**Package of interventions**			
Jiusitthipraphai, 2015^55^	12 weeks (2.8 months): mean scores on oral contraceptive self-efficacy (OCSE) and oral contraceptive used behavior (OCUB) of study group were higher than control group with a statistical significance (*P*<.001)		
Guo, 2022^23^Huber-Krum, 2020^24^Pradhan, 2019^25^Puri, 2021^26^	12 months: use of modern contraception (0.04; 95% CI=0.00, 0.10)(Huber-Krum)	IUD insertion in immediate postpartum period:Intervention increased PPIUD uptake by 4.4% (95% CI=2.8%, 6.4%]). The adherence-adjusted estimate implies that receiving counseling due to the intervention increases uptake of PPIUD by around 17% (95% CI=4%, 40%).(Pradhan)At 1 year:Short-acting contraception: Y1 (0.02, 95% CI=−0.02, 0.07, *P*>.05)Long-acting contraception: Y1 (0.03, 95% CI=0.01, 0.05, *P*<.05)PPIUD: Y1 (0.03, 95% CI=0.02, 0.04, *P*<.05)Non-postpartum IUD LARC: Y1 (−0.00, 95% CI=−0.01, 0.01, *P*>.05)Sterilization: Y1 (−0.01, 95% CI=−0.02, −0.00, *P*<.05)24 months:Short-acting contraception: Y2 (−0.01, 95% CI=−0.04,0.02), *P*>.05)Long-acting contraception: Y2 (0.02, 95% CI=−0.00, 0.04), *P*>.05)PPUID: Y2 (0.02, 95% CI=0.01, 0.03,*P*<.05)Non-PPIUD LARC: Y2 (−0.01, 95 %CI=−0.02, 0.01, *P*>.05)Sterilization: Y2 (−0.01, 95% CI=−0.02, 0.00, *P*>.05)(Huber-Krum)	At 24 months: use of modern contraception (0.00; 95% CI=−0.04, 0.4) (Huber-Krum)Women counseled in either the pre-discharge period (aOR 0.86; 95% CI=0.80, 0.93) or in the post-discharge period (aOR 0.86; 95% CI=0.79, 0.93) were less likely to have an unmet need in the postpartum period compared to women with no counseling^a^; women who received counseling in both the pre- and post-discharge period were 27% less likely than women who had not received counseling to have unmet need (aOR 0.73; 95% CI=0.67, 0.80). (Puri)The adjusted probability of having incident pregnancy was 0.7 percentage points (95% CI=−3.0, 1.4) lower among women in the intervention group than among women in the control group. (Guo)
Karra, 2019^27^		Assessed choice not insertion: 4.1% of women choosing PPIUD prior to the intervention compared to 9.8% of women choosing PPIUD after the rollout of the intervention (0.027; 95% CI=0.000, 0.054).The adherence-adjusted estimate implies that receiving counseling due to the intervention increases uptake of PPIUD by around 8.9% [95% CI=2.7%, 15%].	
Pearson, 2020^28^		Assessed choice not insertion: Increased choice of PPIUD by 6.3% (95% CI=2.3%, 8.0%).The adherence-adjusted estimate implies that receiving counseling due to the intervention increases uptake of PPIUD by around 31.6% (95% CI=24.3%, 35.8%).	
Tran, 2019^29^	12 months: prevalence of modern contraceptive methods in the intervention arm was about twice that of the control arm (55% vs 29%, aPR: 1.79, 95% CI=1.30, 2.47). Also, significant changes in modern contraceptive use were observed at 6 weeks and 6 months.	At 12 months: In the intervention group, increased use of LARCs (aPR: 1.66; 95% CI=1.17, 2.35) and short-acting methods (aPR: 2.01; 95% CI=1.18, 3.43) was observed.Also, significant changes were observed in LARC use at 6 months and in use of short-acting methods at 6 weeks and 6 months.	
Tran, 2020^30^	12 months: prevalence of modern contraceptive methods in the intervention arm was not significantly different from the control group (aPR: 1.58; 95% CI=0.74, 3.38).No difference was observed also at 48 hours, 1 week, 6 weeks, 6 months.	Significant change was observed in use of implants (long-acting) at 6 weeks, 6 months, 12 months.	
Jarvis, 2018^37^	Within 12 months (timing unclear): FP use among all nonpregnant womenModern FP Use OR (95% CI)/aOR(95% CI)Arm 1 (quality): 0.4(0.2,0.8)/0.4(0.2,0.9) P<.05 for bothArm 2 (free): 1.2(0.7,2.0)/0.9(0.5,1.8)Arm 3 (free/quality): 2.3(1.4,3.9) *P*<.005/2.3(1.2,4.3) *P*<.05Control=reference	Among all nonpregnant women:Modern FP use, excluding condomsArm 1: 0.8(0.4,1.7)/1.4(0.6,3.2)Arm 2: 3.2(1.8,5.8) *P*<.001/3.2(1.4,7.2) *P*<.005Arm 3: 6(3.4,10.7)/8.6(3.9,19.0) *P*<.001 for bothLARC UseArm 1: 2.1(0.8,5.4)/2.9(1.1,7.9)Arm 2: 6.3(2.8,14.2)/5.6(2.3,13.7) *P*<.001 for bothArm 3: 8.2(3.7,18.4)/8.4(3.4,20.6) *P*<.001 for bothImplant useArm 1: 1.7(0.6,4.8)/2.3(0.8,6.9)Arm 2: 7.0(3.0,16.4)/5.7(2.2,14.4) *P<.*001 for bothArm 3: 6.8(2.9,16.0)/5.6(2.2/14.4) *P*<.001 for both	
Karra, 2022^44^		At 24 months: Use of long-acting methods increased by 5.4% (95% CI=0.020, 0.089). Use of implants increased by 4.3% (95% CI=0.011, 0.075). No change in use of injectables (0.00088 (95% CI=−0.039, 0.040).	At 24 months: contraceptive use increased in intervention group by 5.9% (95% CI=0.024, 0.094).Intervention group’s hazard of pregnancy was 43.5% lower 24 months after the index birth (based on a hazard rate of 0.565 (95% CI=0.387, 0.824).
Espey, 2021^45^		Over the 15-month intervention period, providers at our intervention facilities inserted 83.5 PP implants per month (SD=51.9) and 224.8 PPIUDs per month (SD=75.3). Notably, prior to our intervention, only 30 PP implant insertions per month and 8 PPIUD insertions per month occurred in our selected facilities.	Receiving more promotions was associated with client uptake for PP implants (test for trend, X2=65.8, *P*<.0001) and PPIUDs (test for trend, X2=26.9, *P*<.0001). Of the 12,068 women who received our intervention and delivered at a study facility, 1252 chose a PP implant (10.4% uptake), 3372 chose a PPIUD (27.9% uptake), and 7444 declined a postpartum LARC method (61.7% non-uptake)
Wu, 2020^51^	Within 12 months: Use of any modern contraceptive method increased from 29% pre-intervention to 46% post-intervention (*P*<.0001).The adjusted OR for any modern contraceptive use of women in the post-intervention group as compared to pre-intervention group was 2.3 (95% CI=1.7, 3.1; *P*<.0001).	With respect to method mix, use of LAM, injectables, and implant increased significantly. Condom use decreased significantly from 4.5% to 1.6% (*P*=.01).	
Ahmed, 2015^53^	12 months: cumulative probability of adopting any modern contraceptive method=65.9% in intervention and 39.1% in comparison arm.CPR**=**42% in intervention and 27% in comparison (*P*<.001).	In intervention arm, higher acceptance of oral contraceptives (aHR=1.33, *P*<.001), condoms (aHR=3.39, *P*<.001), and reduced acceptance of traditional methods (aHR=0.59, *P*=.001).No difference in adoption of injectables and female sterilization. Low acceptance of IUDs in both groups (0.6% in intervention vs. 1.3% in control)Higher use of LAM in intervention arm: 3 months – 23% vs. 0%; 6 months – 12% vs. 0%; no use in either arm at 12 months or 24 months (not reported in article table).	24 months:Cumulative probability of adopting any modern contraceptive method=76.6% in intervention and 54.5% in comparison armThe hazard of all-method adoption was higher in the intervention arm than in the comparison arm (adjusted hazard ratio=2.57, *P*<.001; excluding LAM: aHR=1.51, *P*<.001).CPR=46% in intervention and 35% in comparison (*P*<.001).Continuation rates for first 12 months after adoption show that continuation of oral contraceptives was not significantly (in multivariate analysis) higher in intervention arm [aHR=0.81]; continuation rate of IUDs/implants was higher in intervention arm (85.3%) than in the control arm (59.0%) but was not significantly different in the multivariable model [aHR:0.32). Continuation rates of other methods were not statistically significant.After discontinuation (n=745), 34% of LAM users switched to oral contraceptives, 21% to condoms, 12% to injectables, 1% to IUDs/implants, and 2% to sterilization; 26% remained nonusers at 24 months
Cooper, 2016^56^	Up to 11 months: Effect statistically insignificant for mothers with children 11 months or younger.Up to 24 months: overall, there was a decline in modern contraceptive use over the study period. However, intervention might still have positive effect (In Upper Egypt: OR=1.45, *P*<.001; in Lower Egypt: OR=1.29, *P*<.05).	Use of LARCs generally decreased in intervention and comparison sites over the study period. Measured LAM incorrectly as a breastfeeding method, limiting the ability to interpret this indicator.	When stratifying by children’s age, effect only statistically significant in women with children 12-24m (these are the women that were hardly exposed to antenatal visits)Positive effect on (lower) risk of pregnancy in both Lower (OR=0.40, *P*<.001) and Upper Egypt (OR=0.67, *P*<.001)The intervention appears to have had a positive effect on knowledge of optimal birth spacing in Upper Egypt (OR=1.68, *P*<.001); negative effect on same outcome in Lower Egypt (OR=0.55, *P*<.001)Positive effect on joint contraceptive decision making in both Lower and Upper Egypt
Buser, 2021^47^	Women who gave birth in the last 13 months: aOR contraceptive use (also referred to as avoiding pregnancy/actively avoiding pregnancy) among those who used the Core MWH Model compared to those who did not: 1.33 (1.08–1.63, *P*<.05)		
Maru, 2017^49^	12 months: postpartum contraceptive prevalence increased from 19.0% to 46.5% (difference=27.5%, 95% CI=20.8% to 34.2%, *P*<.001).		
**Training intervention**			
Dhital, 2021^50^			In the adjusted model, a 25-fold increase in FCHV knowledge had been observed at the post-test [aOR=25.4 (CI=12.6, 50.2), *P*<.001], and at 1-year post-intervention, it remained approximately 11-fold higher [aOR=10.7(CI=6.3, 18.1), *P*<.001] as compared to the pre-intervention phase.

Abbreviations: aHR, adjusted hazard ratio; ANC, antenatal care; aOR, adjusted odds ratio; aPR, adjusted prevalence ratio; aRR, adjusted relative risk; CI, confidence interval; CPR, contraceptive prevalence rate; FCHV, female community health volunteer; FP, family planning; IUD, intrauterine device; LAM, lactational amenorrhea method; LARC, long-acting reversible contraceptive; MWH, maternity waiting home; OR, odds ratio; PP, postpartum; PPIUD, postpartum intrauterine device; RD, risk difference; RR, relative risk; SD, standard deviation; SMS, short message service.

a Information regarding outcomes largely taken verbatim from the text.

#### Contraceptive Use Outcomes: General Patterns in Effect Direction and Size

Of the 24 studies (77%) that reported on voluntary postpartum contraceptive use within 1 year after birth, 18 reported a positive intervention effect (75%). Of these 18, 9 (50%) were package interventions,^23^^–^^26^^,^^29^^,^^30^^,^^37^^,^^47^^,^^49^^,^^51^^,^^53^^,^^56^ 4 (22%) were counseling interventions,^36^^,^^38^^,^^39^^,^^43^ 3 (17%) were educational interventions,^35^^,^^42^^,^^54^ 1 (6%) was digital,^33^ and 1 (6%) was financial.^34^ Nine of the 14 package interventions (64%), 3 of the 5 educational interventions (60%), 4 of the 7 counseling interventions (57%), 1 of the 2 digital interventions (50%), and 1 of the 2 financial interventions (50%) reported a positive effect on the primary outcome. The 1 training intervention did not report on the primary outcome.

Of the 24 studies that reported on voluntary postpartum contraceptive use within 1 year after birth, 18 studies reported a positive intervention effect.

While the measures of effect captured and the effect sizes were wide ranging (e.g., relative risk 1.10 to odds ratio [OR] 6.69),^36^^,^^42^ the largest, most consistent effects were seen in the package interventions. Among the package interventions where the main outcome was reported (n=10), 9 (90%) reported a positive intervention effect (this compares to 4 of the 6 counseling interventions where the main outcome was reported (67%); 3 of the 5 educational interventions (60%); 1 of the 2 digital interventions (50%) and 100% of the digital interventions (n=1)). Two package intervention studies reported immediate postpartum contraceptive uptake,^25^^,^^45^ all of which reported a positive effect of the intervention. Among the 6 studies where the main outcome was reported but where there was no significant effect observed,^30^^,^^31^^,^^40^^,^^41^^,^^46^^,^^48^ 1 was a package intervention, 2 were counseling interventions, 2 were educational group sessions, and 1 was a digital intervention.

Four studies^23^^–^^26^^,^^44^^,^^53^^,^^56^ reported on postpartum contraceptive use beyond 1 year after birth; 3 of these reported a statistically significant intervention effect.

Twenty-two studies reported on the use of specific methods of contraception (Table 2, column title Use of specific methods of contraception), with 13^24^^,^^25^^,^^28^^–^^30^^,^^33^^,^^36^^,^^37^^,^^45^^,^^46^^,^^51^ reporting the intervention had a positive effect on use of at least 1 of the following methods: PPIUD, implants, permanent methods, or use of highly effective methods. Five studies reported on LAM and 3 on exclusive breastfeeding. Three of the studies increased use of LAM in the intervention group, 1 decreased it, and 1 showed no use of LAM whatsoever. The studies on exclusive breastfeeding all showed increased use. A further 2 studies trained on the correct use of LAM but did not report results because of measurement problems. Four studies reported on contraceptive continuation (continuation for the first 12 months after adoption, discontinuation at 6 months, continuation among women starting contraception at 10 weeks postpartum, and stopping contraception at 6.5 months),^31^^,^^33^^,^^40^^,^^53^ none of which reported a statistically significant intervention effect.

#### Randomized Controlled Trials

Of the 24 studies that reported on the main outcome of interest, 11 were randomized controlled trials (RCTs) and 13 were quasi-experimental designs. Of the 11 RCTs, 5 used cluster or stepped-wedge designs^23^^–^^26^^,^^29^^,^^30^^,^^35^^,^^46^ and 6 were individually randomized trials.^31^^,^^33^^,^^34^^,^^36^^,^^38^^,^^39^ Utilizing our quality appraisal tool (Table 3), we found that all but 1^31^ of these 11 RCTs described the source population well and included an eligible population that appeared representative of the source population (Unger^31^ was unclear on both of these criteria). All 11 RCTs described the intervention and comparator well. However, only 4^30^^,^^31^^,^^35^^,^^39^ had a low risk of bias regarding the random sequence generation. Two^38^^,^^46^ had a high risk of bias, and the sequence generation was unclear in the remaining 5 trials. This means that in 7 of the 11 RCTs, there was likely a lack of comparability between the trial groups. The trials with the low risk of bias in sequence generation had moderate differences in effect between the groups: increased contraceptive adoption at 12 months (risk difference: 7.2%, 95% confidence interval [CI]=2.6, 12.9, *P*=.034)^35^; use of modern contraception at 6 months postpartum: 57.4% vs. 35.4%, *P*=.002^39^; prevalence of modern contraceptive methods at 12 months (adjusted prevalence ratio: 1.58, 95% CI=0.74, 3.38)^30^; and increased contraceptive use in both intervention arms (1-way SMS: 72% and 2-way SMS: 73%; *P*=.03 and .02, respectively, vs. 57% control; however, this difference was not significant when correcting for multiple comparisons).^31^

**TABLE 3.** Quality Appraisal

**Author**	**Quasi/RCT**	**Well-Described Source Population?**	**Eligible Population Representative of Source Populations?**	**Well-Described Intervention(s)?**	**Well-Described Comparator(s)?**	**Random Sequence Generation Risk of Bias?**	**Baseline (or Group) Characteristics Similar Risk of Bias?**	**Outcome Data Completeness Risk of Bias?**	**Adequate Analytical Methods?**
**Counseling interventions**									
Ndegwa^32^	Quasi	Unclear	Unclear	No	Yes	Unclear	High	Low	No
Adanikin^39^	RCT	Yes	Yes	Yes	Yes	Low	Low	Low	Yes
Camara^43^	Quasi	Yes	Yes	Yes	Yes	Unclear	Low	Low	No
Ayiasi^46^	cRCT	Yes	Yes	Yes	Yes	High	High	Unclear	No
Keogh^40^	Quasi	Yes	Yes	Yes	Yes	NA	High	High	Yes
Abdulkadir^38^	RCT	Yes	Yes	Yes	Yes	High	Low	Low	Yes
Daniele^36^	RCT	Yes	Yes	Yes	Yes	Unclear	Low	Low	Yes
**Digital interventions**									
Unger^31^	RCT	Unclear	Unclear	Yes	Yes	Low	Low	Low	Yes
Harrington^33^	RCT	Yes	Yes	Yes	Yes	Unclear	High	Low	Yes
**Educational interventions**									
Sebastian^54^	Quasi	Yes	Yes	Yes	Yes	Unclear	Low	Low	Yes
Maldonado^35^	cRCT	Yes	Yes	Yes	Yes	Low	Low	Low	Yes
Bang^41^	Quasi	Yes	Yes	Yes	Yes	NA	High	Unclear	Yes
Lori^42^	Quasi	Yes	Yes	Yes	Yes	NA	Low	High	Yes
Sarnquist^48^	Quasi	Yes	Yes	Yes	Yes	NA	Low	Low	Yes
**Financial interventions**									
McConnell^34^	RCT	Yes	Yes	Yes	Yes	Unclear	Low	High	Yes
Engineer^52^	cRCT	Yes	Yes	Yes	Yes	Unclear	Low	Unclear	Yes
**Package of interventions**									
Jiusitthipraphai^55^	Quasi	Yes	Yes	Yes	Yes	NA	High	Low	Yes
Guo^23^Huber-Krum^24^Pradhan^25^Puri^26^	cRSWD	Yes	Yes	Yes	Yes	Unclear	Low	Low	Yes
Karra^27^	cRSWD	Yes	Yes	Yes	Yes	Unclear	Low	Low	Yes
Pearson^28^	cRSWD	Yes	Yes	Yes	Yes	Unclear	Low	Low	Yes
Tran	cRCT	Yes	Yes	Yes	Yes	Unclear	High	Low	Yes
Tran	cRCT	Yes	Yes	Yes	Yes	Low	Low	Low	Yes
Jarvis	Quasi	Yes	Yes	Yes	Yes	NA	High	High	Yes
Karra	RCT	Yes	Yes	Yes	Yes	Low	Low	High	Yes
Espey	Quasi	NR	NR	Yes	NA	NA	NA	NA	Unclear
Wu	Quasi	Yes	Yes	Yes	Yes	NA	Low	Low	Yes
Ahmed	Quasi	Yes	Unclear	Yes	Yes	NA	High	Unclear	Yes
Cooper	Quasi	Yes	Yes	Yes	Yes	NA	Low	Unclear	Yes
Buser	Quasi	Yes	Yes	Yes	NA	NA	NA	NA	Yes
Maru	Quasi	Unclear	Unclear	Unclear	NA	NA	NA	NA	Yes
**Training intervention**									
Dhital	Quasi	Yes	Yes	Unclear	NA	NA	NA	Low	Yes

Abbreviations: cRCT, cluster randomized controlled trial; cRSWD, cluster-randomized stepped-wedge design; NA, not applicable; NR, not reported; RCT, randomized controlled trial.

#### Intervention Descriptions

The 18 studies reporting a positive intervention effect on the main outcome were also heterogeneous regarding intervention type. Nine of these studies^23^–26^,^^29^^,^^37^^,^^47^^,^^49^^,^^51^^,^^53^^,^^55^^,^^56^ a “package”-type intervention and 6 of these 9 (66%) were characterized as “multifaceted” with several interacting intervention components. All but 1 (Tran et al.^29^) of the multifaceted interventions that reported PPFP use within a year reported a positive intervention effect. Although they did not report on the primary outcome of interest, the 7-component package described by Karra et al.^27^ (FIGO’s PPIUD intervention, described later) to institutionalize immediate PPIUD services as part of routine ANC and delivery-room services reported a doubling of women choosing PPIUD at 24 months after the rollout of the intervention (from 4.1% before to 9.8% after).

The Jarvis et al.^37^ quasi-experimental study evaluated a 3-armed intervention that provided (1) free FP; (2) a “quality inputs” intervention consisting of clinical training and provision of PPIUD insertion equipment, training staff on WHO’s Medical Eligibility Criteria for contraceptive use, and a systematic FP screening and referral tool; (3) a combination of free FP and quality inputs (1 and 2). The authors reported benefits in the quality and free arms (arms 1 or 2), but the greatest effect in the free plus quality arm (arm 3) long-acting, reversible contraceptive (LARC) use within 12 months postpartum (OR=8.4; 95% CI, 3.4, 20.6). However, this study had a high risk of bias because several baseline group characteristics were dissimilar between the intervention arms compared to the control group, and the outcome data were incomplete.

The 4 reports in Nepal^23^^–^^26^ evaluated the 7-component intervention through another cluster stepped-wedge trial aimed at increasing PPIUD.^25^ This FIGO-designed intervention consisted of (1) informational workshops for female community health volunteers and general hospital staff; (2) training maternity care providers in PPFP counseling, PPIUD insertion, and management of complications; (3) PPFP leaflets distributed during counseling; (4) informational wall chart and video displayed in the hospital waiting area; (5) provision of Kelley’s forceps for IUD insertion and provision of IUDs; (6) designated service provider in each hospital as facility coordinator for the program; and (7) regular monitoring of counseling and insertion data. The results showed an increase in PPIUD uptake in the immediate postpartum (adjusted risk difference of 4% (95% CI=3, 6).

The Huber-Krum et al.^24^ analysis of the Nepal FIGO package intervention looked at the effect of the intervention on modern contraceptive prevalence and method mix rather than just on PPIUD insertion immediately after birth. This analysis found an adjusted risk difference of 4% (95% CI=−0.00, 0.10) more uptake of modern methods at 1 year postpartum, demonstrating a small and borderline statistically significant effect. They also found the 4% increase in PPIUD uptake in the intervention group observed by Pradhan et al. was somewhat diminished to 3% at 12 months postpartum but remained statistically significant (95% CI=0.02, 0.04). By 24 months, most differences between the intervention and comparison groups had disappeared, except for PPIUD, which had an adjusted risk difference of 2% (95% CI=0.01, 0.03).

The Karra et al.^44^ package intervention consisted of 4 services over a 2-year period: up to 6 FP counseling sessions, free transportation to an FP clinic, free FP services, and treatment for contraceptive-related side effects. The authors of this large cluster stepped-edge trial (39,084 women in 6 hospitals) reported that contraceptive use after 2 years of exposure to the intervention increased by 5.9%, mainly through increases in the use of implants.

Tran et al.^29^ conducted a cluster-randomized trial in Burkina Faso evaluated a package of 3 facility-oriented interventions (refresher training of service providers, regularly scheduled and strengthened supportive supervision of providers, enhanced availability of services 7 days a week) and 3 individual-based interventions delivered during third-trimester ANC visits and postnatal care follow-up visits (a PPFP counseling tool, appointment cards for women, and invitation letters for partners). The authors reported that the intervention improved the prevalence ratio of modern contraceptive use at 12 months (adjusted prevalence ratio 1.7, 95% CI=1.3, 2.47). The intervention had its greatest effect in promoting uptake of FP at 6 weeks, with an adjusted prevalence ratio of 3.88 (95% CI=1.46, 10.35), but the effect reduced somewhat over time, with the 6-month adjusted prevalence ratio at 2.31 (95% CI=1.44, 3.71).

Cooper et al.^56^ evaluated a package intervention in Egypt called the SMART project, using a controlled before-after design. SMART aimed to decrease child malnutrition through an “integrated, community-based reproductive and maternal and child health intervention package.” SMART was implemented during a political transition in Egypt and, as such, FP was deemphasized in the beginning to respond to the political and social climate. The intervention consisted of 3 overarching components, within which existed various activities: one-to-one and group counseling, training health care workers, and mobile clinics. The counseling covered the benefits of FP, healthy timing and spacing of pregnancies, postpartum return to fertility and pregnancy risk after childbirth, and LAM and transition to other modern contraceptive methods. The project coordinated with local health directors to advocate for the availability of FP commodities at government health facilities and mobile clinics. Women in the intervention group were more likely to use a modern method of contraception (Upper Egypt: OR=1.45, *P*<.001; Lower Egypt: OR=1.29, *P*<.05). However, the difference was not statistically significant for mothers with children aged 11 months or younger (Upper Egypt: OR=1.13, *P*>.05; Lower Egypt: OR=1.20, *P*>.05), which authors said could be due to differences in the duration of exposure to the program during the antenatal and postpartum periods among mothers with children aged 11 months or younger compared to mothers with older children.

The package intervention evaluated by Wu et al.^51^ included active pregnancy identification, home-based ANC and postnatal care counseling and care coordination, patient-centered contraceptive counseling, group ANC, and home-based childcare and counseling. The intervention was longitudinal, starting in the antenatal period and continuing through the first year postpartum. Counseling topics covered recommendations and reasons for birth spacing, contraceptive efficacy, contraindications, and timing for postpartum method initiation. Training materials emphasized best practices for contraceptive counseling (e.g., shared decision-making, patient autonomy, and guidance on potential side effects). This uncontrolled before-after study reported that use of any modern contraceptive method increased from 29% pre-intervention to 46% post-intervention (*P*<.0001). Participants spoke about how much they valued the continuity of care and relationships with the providers, which the authors said likely helped address women’s changing contraceptive needs during the first year postpartum.

Twelve studies used a counseling (n=7)^32^^,^^36^^,^^38^^–^^40^^,^^43^^,^^46^ or an education (n=5) intervention,^35^^,^^41^^,^^42^^,^^48^^,^^54^ and 2^31^^,^^33^ used an SMS intervention. Thirteen of these 14 measured the outcome of interest. Eight of these 13 (62%) showed a statistically significant impact on uptake of contraception, although most assessed use well before 12 months postpartum. In general, the magnitude of the intervention was strongest the shorter the time period examined, although in 1 case,^43^ the effect was greater at 9 months than at 6 months postpartum. The magnitudes of the risk differences for modern/effective methods of contraception ranged from -0.1% at 3 months,^48^ 17.5%^38^ at 16 weeks, 22%^39^ at 6 months, 7.1%^35^ and 6.4%^36^ at 8 months, 4.6%^43^ and 24.2%^54^ at 9 months, 40.1%^42^ at 1 year, no significant differences at 6–15 months,^40^ and 26.3% in a survey 2 years (midpoint 12 months) after baseline.^41^

### Quality Appraisal Overview

Most studies described their source population well (n=27, 87%), defined an eligible population that was representative of the source population (n=26, 84%), described the intervention well (n=28, 90%), and described the comparator well (27/27, 100%) (Table 3). Among the RCTs (n=16), only 5 (31%) had a low risk of bias regarding the random sequence generation, 2 had a high risk of bias (13%), and 9 (61%) had an unclear risk of bias. Among the 26 studies where it was applicable, 18 (69%) had a low risk of bias regarding the comparability of the baseline (or group) characteristics.

## DISCUSSION

### Summary of Findings

We double-screened 771 records and included 34 reports on 31 studies in the review. This is a substantial increase from the 16 studies identified by Cleland et al. for the period before 2012.^4^ Our study is the first review to capitalize on the increasing recent research and the growing interest in PPFP among donors and policymakers. The majority of included studies (n=21) were published since 2018. Most (n=21) were conducted in sub-Saharan Africa, with half of the studies enrolling pregnant women (n=17). Twelve studies had interventions in the antenatal period only.

Approximately half of the study designs (n=16) were RCTs, and half (n=15) were quasi-experimental. The quality appraisal showed generally positive results, with most studies describing their source population, representativeness, intervention, and comparator well. In the trials, random sequence generation could have been improved in many studies. Most evaluations (n=24) were conducted in health facility settings (with 2 also in community settings); 7 were in community settings only (these findings are not very dissimilar to those reported by Cleland et al.). Interventions were heterogeneous, with the distinction between counseling and education interventions not always clear.

Twenty-four of the 31 studies reported on the main outcome of interest in this review (postpartum contraceptive use within 1 year of birth), with 18 of these 24 studies reporting a positive intervention effect—compared to 9 of 16 of the older studies reviewed by Cleland et al. In general, the magnitude of the effect of the intervention was stronger the shorter the time period examined; studies that looked over several time points saw a decrease in the effect over time, although in 1 case, the effect was greater at 9 months compared to 6 months postpartum. This could suggest the effect of the interventions encouraged women to start earlier, but that over time, even women in the nonintervention groups take up contraception. It could also suggest that while women who receive the intervention start a method earlier, they may also discontinue. Interventions may also encourage women to adopt specific methods but not usage overall. For example, interventions that target uptake of LARC uptake, which have a higher continuation rate at later time points may not be comparable to interventions that do not specifically promote a long-acting method. Given that effect sizes tended to decrease over time, the different times at assessment of contraceptive usage could be expected to greatly affect the comparability of the studies.

Although the studies in this systematic review were heterogeneous, the findings suggest that the interventions that included a multifaceted package of initiatives appeared to be more likely to have a positive effect. By contrast, interventions with minimal counseling did not appear to be as effective. Only 2 of the 10 multifaceted packages were delivered during the antenatal period only, and given the relatively small number of studies for any given type of intervention, it is challenging to draw additional conclusions beyond the broad ones made earlier. For the multifaceted package intervention, assessment of the effect of the individual components of the intervention is not possible as they were evaluated overall. Future studies could assess the influence of the different intervention components on each other to understand how they work together to affect postpartum contraceptive uptake.

The findings suggest that the interventions that included a multifaceted package of initiatives appeared to be more likely to have a positive effect.

To aid in the comparability of findings across studies, we recommend methodological improvements. Future evaluations of ANC interventions to increase postpartum contraceptive use could measure use at standardized key time points (e.g., at 3, 6, and 12 months). Moreover, future implementation research needs to more specifically address the perceived susceptibility (i.e., how protected against pregnancy someone thinks they are when using LAM) and actual susceptibility (i.e., how protected against pregnancy they actually are) when using LAM, perhaps by looking at waiting time to conception, and on how reliance on LAM and transitioning from LAM can affect timing of uptake of other methods. Future research could also focus on documenting more definitive results on counseling interventions in the context of group ANC versus individual ANC and on the most promising packages that consider specific contexts and health service gaps.

### Strengths and Limitations

Our search incorporated all LMICs in scope, was built on similar preexisting reviews, and reviewed 3 databases. All records were double-screened, with reviewers masked to other reviewers’ decisions. Our synthesis approach was robust, and there appeared to be enough information to assess eligibility of the studies. The quality of the included studies was generally good. The measures taken to minimize bias in the review included double-screening and double extraction; however, the quality appraisal was conducted by 1 person. Compared to the older reviews, which primarily involved counseling, the studies covered a wider range of intervention approaches that focused on, for example, training providers, client voucher provision, systems strengthening, and pay for performance. While this review did not produce clear evidence for the effectiveness of these “new” approaches, it indicates that novel approaches are being considered and highlights the need for further evaluation of them.

The heterogeneity in interventions, population, outcomes, and time period when outcomes were assessed made it difficult to identify themes and precluded conducting a meta-analysis. This means the body of evidence may be too small to make definitive recommendations for any given approach. Moreover, we did not fully consider all the relationships between the intervention characteristics and all the outcomes or how contextual variables could affect the outcomes. For example, a systematic review by Gahungu et al.^57^ on unmet need for PPFP among women in 5 sub-Saharan African countries identified individual-level factors associated with unmet need, such as low perceived risk of pregnancy, resumption of menses and sexual activity, and factors on the household level, such as husband’s approval and spousal discussion about FP. Few studies in our review reported on these variables, making it difficult to assess potential confounders or contextual differences explaining the magnitude of effects in the different studies. Studies promoting multiple methods (e.g., LARCs and LAM) might see declines in some methods relative to others, whereas studies promoting only 1 method were more likely to see use of this method increase.

There was an additional limitation related to the timing of the intervention delivery. We included interventions that were not exclusively delivered during the antenatal period. The benefit of this is that the review results present a more complete picture of the range of antenatal interventions to increase contraceptive use following birth rather than being restricted to the 12 studies that described antenatal-only interventions. Indeed, it can be argued that an intervention integrated into a continuum of pregnancy care is desirable. The limitation of this, though, is that any effect of an intervention delivered across the different periods cannot be attributed to its delivery during the antenatal period and that comparisons between the periods cannot be made. However, attribution to the antenatal period or other period was not the aim of this review.

## CONCLUSIONS

The studies in this systematic review were heterogeneous, and the number of studies with any given approach was small. Nevertheless, the findings suggest that interventions that included a multifaceted package of initiatives appeared to be more likely to have a positive effect and that those with minimal counseling did not appear to be effective. To help avert short interpregnancy intervals and improve health outcomes for mothers and babies, funders, policymakers, and providers may consider strengthening their support for multifaceted ANC interventions delivered over multiple time points that promote voluntary informed choice regarding postpartum contraception.

## Supplementary Material

GHSP-D-24-00059_supplement.docx

## Notes

**Peer Reviewed**

**First Published Online:** September 16, 2024.
