# Supplementary material for: Antenatal Care Interventions to Increase Contraceptive Use Following Birth in Low- and Middle-Income Countries: Systematic Review and Narrative Synthesis
Source: Glob Health Sci Pract. 2024 Oct 29;12(5):e2400059. doi: 10.9745/GHSP-D-24-00059 (PMC11521549; doi:10.9745/GHSP-D-24-00059)
Supplement: GHSP-D-24-00059_supplement.docx [file GHSP-D-24-00059_supplement.docx]

| **Ovid MEDLINE(R) ALL <1946 to July 29, 2031>** | |
| --- | --- |
|  |  |
| 1 | (postpartum or post-partum or postnatal or post-natal or puerperium or "after deliver*" or "after childbirth" or "after birth*" or "since birth*" or "following deliver*" or "following childbirth" or "following birth*").mp. or exp Postpartum Period/ [mp=title, book title, abstract, original title, name of substance word, subject heading word, floating sub-heading word, keyword heading word, organism supplementary concept word, protocol supplementary concept word, rare disease supplementary concept word, unique identifier, synonyms] |
| 2 | (contracept* or "family planning" or "birth control" or "fertility control" or "birth regulation" or "fertility regulation" or (birth* adj2 spac*) or (child adj2 spac*) or (childbirth adj2 spac*) or (birth* adj2 timi*) or (pregnanc* adj2 timi*) or (pregnanc* adj2 spac*) or (childbirth adj3 interval*) or (birth* adj3 interval*) or (pregnanc* adj3 interval*) or (conce* adj2 interval*)).mp. or exp Contraception/ or exp Contraceptive Devices/ or depo? Medroxyprogesterone.mp. or Depo-Provera.mp. or Sayana Press.mp. or IUD.mp. or IUCD.mp. or IUS.mp. or intra?uterine device*.mp. or intra?uterine system*.mp. or oral contraceptive pill*.mp. or hormonal contraceptive pill*.mp. or birth control pill.mp. or mini?pill.mp. or progesterone?only pill.mp. or emergency contraceptive.mp. or cervical cap.mp. or cervical caps.mp. or vaginal diaphragm*.mp. or vaginal ring*.mp. or implant*.mp. or subdermal implant*.mp. or implanon.mp. or jadelle.mp. or norplant*.mp. or sino?implant.mp. or sterili?ation.mp. or vasectomy.mp. or contraception behavio?r.mp. or long-acting reversible contraception.mp. or LARCS.mp. or family planning/ or birth control/ or contraception/ or Contraception Behavio?r/ or Family Planning Services/ [mp=title, book title, abstract, original title, name of substance word, subject heading word, floating sub-heading word, keyword heading word, organism supplementary concept word, protocol supplementary concept word, rare disease supplementary concept word, unique identifier, synonyms] |
| 3 | (method* or interven* or meet* or consult* or advi* or counsel* or visit* or session* or educat* or communicat* or involve* or communit* or facilit* or service* or deliver* or provi* or program* or campaign* or scheme* or outreach*).mp. or exp intervention studies/ or (Family planning counsel?ing or family planning information or family planning advice or contraceptive counsel?ing).mp. [mp=title, book title, abstract, original title, name of substance word, subject heading word, floating sub-heading word, keyword heading word, organism supplementary concept word, protocol supplementary concept word, rare disease supplementary concept word, unique identifier, synonyms] |
| 4 | (ANC or PNC or ((antenatal or prenatal or pre?natal or pregnancy) and care)).mp. or exp Prenatal care/ [mp=title, book title, abstract, original title, name of substance word, subject heading word, floating sub-heading word, keyword heading word, organism supplementary concept word, protocol supplementary concept word, rare disease supplementary concept word, unique identifier, synonyms] |
| 5 | (trial* or control* or randomi* or evaluat* or prospective or longitudinal or blind* or single-blind* or "single blind*" or double-blind* or "double blind*" or "before and after" or crossover or cross-over or cluster* or placebo or arm or arms or "quasi?experiment*" or "pre?post" or meta-analysis or synthesis or literature or published or extraction or search or review*).mp. or (quasiexperiment$2 or quasi experiment$2).ti,ab. or exogenous variation$1.ti,ab. or natural experiment$2.ti,ab. or Matched controls.ti,ab. or Counterfactual outcome$1.ti,ab. or Rubin causal model$1.ti,ab. or potential outcomes model$1.ti,ab. or (Identification adj (strategy or assumptions or conditions)).ti,ab. or (conditional adj (independence or ignorability)).ti,ab. or unobserved heterogeneity.ti,ab. or Unconfoundness.ti,ab. or Confounding.ti,ab. or (instrumental variable$1 adj (analysis or analyses or estimation)).ti,ab. or (overidentification or overidentifying).ti,ab. or regression discontinuity analys$2.ti,ab. or ((balancing or imbalance or balanced or imbalanced) adj3 covariates).ti,ab. or interrupted time series.ti,ab. or difference studies.ti,ab. or (controlled adj3 before adj3 after).ti,ab. or ((exact or score or genetic or nearest neighbor or nearest neighbour or caliper or radius or kernel density or blocking or stratification of interval) adj3 matching).ti,ab. or (Inverse probability weight$ adj4 estimat$).ti,ab. or (doubly robust adj4 (regression or estimate$)).ti,ab. or ((treatment or switching or selection or selectivity) adj3 regression).ti,ab. or (selection model or selectivity model).ti,ab. or (heckit model or heckman sample selection).ti,ab. or selection correction.ti,ab. or two stage residual inclusion.ti,ab. or regression discontinuity.ti,ab. or (sharp design or fuzzy design).ti,ab. or Forcing variable$1.ti,ab. or (difference$1 adj3 difference$1).ti,ab. or (change$1 adj3 change$1).ti,ab. or (Fixed effects and panel data).ti,ab. or full information maximum likelihood.ti,ab. or ((health or economic) adj shock$1).ti,ab. or natural controls.ti,ab. [mp=title, book title, abstract, original title, name of substance word, subject heading word, floating sub-heading word, keyword heading word, organism supplementary concept word, protocol supplementary concept word, rare disease supplementary concept word, unique identifier, synonyms] |
| 6 | (emerging country or emerging countries or emerging nation or emerging nations or emerging population or emerging populations developing country or developing countries or developing nation or developing nations or developing population or developing populations or developing world or less developed country or less developed countries or less developed nation or less developed nations or less developed population or less developed populations or less developed world or lesser developed country or lesser developed countries or lesser developed nation or lesser developed nations or lesser developed population or lesser developed populations or lesser developed world or under developed country or under developed countries or under developed nation or under developed nations or under developed population or under developed populations or under developed world or underdeveloped country or underdeveloped countries or underdeveloped nation or underdeveloped nations or underdeveloped population or underdeveloped populations or underdeveloped world or middle income country or middle income countries or middle income nation or middle income nations or middle income population or middle income populations or low income country or low income countries or low income nation or low income nations or low income population or low income populations or lower income country or lower income countries or lower income nation or lower income nations or lower income population or lower income populations or underserved country or underserved countries or underserved nation or underserved nations or underserved population or underserved populations or underserved world or under served country or under served countries or under served nation or under served nations or under served population or under served populations or under served world or deprived country or deprived countries or deprived nation or deprived nations or deprived population or deprived populations or deprived world or poor country or poor countries or poor nation or poor nations or poor population or poor populations or poor world or poorer country or poorer countries or poorer nation or poorer nations or poorer population or poorer populations or poorer world or developing economy or developing economies or less developed economy or less developed economies or lesser developed economy or lesser developed economies or under developed economy or under developed economies or underdeveloped economy or underdeveloped economies or middle income economy or middle income economies or low income economy or low income economies or lower income economy or lower income economies or low gdp or low gnp or low gross domestic or low gross national or lower gdp or lower gnp or lower gross domestic or lower gross national or lmic or lmics or third world or lami country or lami countries or transitional country or transitional countries or Africa or Asia or Caribbean or West Indies or South America or Latin America or Central America or Atlantic Islands or Pacific Islands or Indian Ocean Islands or Eastern Europe or Afghanistan or Albania or Algeria or Angola or Antigua or Barbuda or Argentina or Armenia or Armenian or Aruba or Azerbaijan or Bahrain or Bangladesh or Barbados or Benin or Byelarus or Byelorussian or Belarus or Belorussian or Belorussia or Belize or Bhutan or Bolivia or Bosnia or Herzegovina or Hercegovina or Botswana or Brasil or Brazil or Bulgaria or Burkina Faso or Burkina Fasso or Upper Volta or Burundi or Urundi or Cambodia or Khmer Republic or Kampuchea or Cameroon or Cameroons or Cameron or Camerons or Cape Verde or Central African Republic or Chad or Chile or China or Colombia or Comoros or Comoro Islands or Comores or Mayotte or Congo or Zaire or Costa Rica or Cote d'Ivoire or Ivory Coast or Croatia or Cuba or Cyprus or Czechoslovakia or Czech Republic or Slovakia or Slovak Republic or Djibouti or French Somaliland or Dominica or Dominican Republic or East Timor or East Timur or Timor Leste or Ecuador or Egypt or United Arab Republic or El Salvador or Eritrea or Estonia or Ethiopia or Fiji or Gabon or Gabonese Republic or Gambia or Gaza or Georgia Republic or Georgian Republic or Ghana or Gold Coast or Greece or Grenada or Guatemala or Guinea or Guam or Guiana or Guyana or Haiti or Honduras or Hungary or India or Maldives or Indonesia or Iran or Iraq or Jamaica or Jordan or Kazakhstan or Kazakh or Kenya or Kiribati or Korea or Kosovo or Kyrgyzstan or Kirghizia or Kyrgyz Republic or Kirghiz or Kirgizstan or Lao PDR or Laos or Latvia or Lebanon or Lesotho or Basutoland or Liberia or Libya or Lithuania or Macedonia or Madagascar or Malagasy Republic or Malaysia or Malaya or Malay or Sabah or Sarawak or Malawi or Nyasaland or Mali or Malta or Marshall Islands or Mauritania or Mauritius or Agalega Islands or Melanesia or Mexico or Micronesia or Middle East or Moldova or Moldovia or Moldovian or Mongolia or Montenegro or Morocco or Ifni or Mozambique or Myanmar or Myanma or Burma or Namibia or Nepal or Netherlands Antilles or New Caledonia or Nicaragua or Niger or Nigeria or Northern Mariana Islands or Oman or Muscat or Pakistan or Palau or Palestine or Panama or Paraguay or Peru or Philippines or Philipines or Phillipines or Phillippines or Poland or Portugal or Puerto Rico or Romania or Rumania or Roumania or Russia or Russian or Rwanda or Ruanda or Saint Kitts or St Kitts or Nevis or Saint Lucia or St Lucia or Saint Vincent or St Vincent or Grenadines or Samoa or Samoan Islands or Navigator Island or Navigator Islands or Sao Tome or Saudi Arabia or Senegal or Serbia or Montenegro or Seychelles or Sierra Leone or Slovenia or Sri Lanka or Ceylon or Solomon Islands or Somalia or Sudan or Suriname or Surinam or Swaziland or Syria or Syrian or Tajikistan or Tadzhikistan or Tadjikistan or Tadzhik or Tanzania or Thailand or Togo or Togolese Republic or Tonga or Trinidad or Tobago or Tunisia or Turkey or Turkmenistan or Turkmen or Tuvalu or Uganda or Ukraine or Uruguay or USSR or Soviet Union or Soviet Socialist Republics or Uzbekistan or Uzbek or Vanuatu or New Hebrides or Venezuela or Vietnam or Viet Nam or West Bank or Yemen or Yugoslavia or Zambia or Zimbabwe or Rhodesia or Developing Countries or Africa or Asia or Caribbean Region or West Indies or South America or Latin America or Central America or Atlantic Islands or Pacific Islands or Indian Ocean Islands or Afghanistan or Albania or Algeria or American Samoa or Angola or "Antigua and Barbuda" or Argentina or Armenia or Azerbaijan or Bahrain or Baltic States or Bangladesh or Barbados or Benin or Belarus or Belize or Bhutan or Bolivia or Bosnia-Herzegovina or Botswana or Brazil or Bulgaria or Burkina Faso or Burundi or Cambodia or Cameroon or Cape Verde or Central African Republic or Chad or Chile or China or Colombia or Comoros or Congo or Costa Rica or Cote d'Ivoire or Croatia or Cuba or Cyprus or Czechoslovakia or Czech Republic or Slovakia or Djibouti or Congo or Korea or Dominica or Dominican Republic or East Timor or Ecuador or Egypt or El Salvador or Eritrea or Estonia or Ethiopia or Equatorial Guinea or Fiji or French Guiana or Gabon or Gambia or Georgia or Ghana or Greece or Grenada or Guatemala or Guinea or Guinea-Bissau or Guam or Guyana or Haiti or Honduras or Hungary or Samoa or India or Indonesia or Iran or Iraq or Jamaica or Jordan or Kazakhstan or Kenya or Korea or Kyrgyzstan or Laos or Latvia or Lebanon or Lesotho or Liberia or Libya or Lithuania or Macedonia or Madagascar or Malawi or Malaysia or Mali or Malta or Mauritania or Mauritius or Melanesia or Mexico or Micronesia or Middle East or Moldova or Mongolia or Montenegro or Morocco or Mozambique or Myanmar or Namibia or Nepal or Netherlands Antilles or New Caledonia or Nicaragua or Niger or Nigeria or Oman or Pakistan or Palau or Panama or Papua New Guinea or Paraguay or Peru or Philippines or Poland or Portugal or Puerto Rico or Romania or Russia or Russia or Rwanda or "Saint Kitts and Nevis" or Saint Lucia or Grenadines or Samoa or Saudi Arabia or Senegal or Serbia or Montenegro or Seychelles or Sierra Leone or Slovenia or Sri Lanka or Somalia or South Africa or Sudan or Suriname or Swaziland or Syria or Tajikistan or Tanzania or Thailand or Togo or Tonga or "Trinidad and Tobago" or Tunisia or Turkey or Turkmenistan or Uganda or Ukraine or Uruguay or USSR or Uzbekistan or Vanuatu or Venezuela or Vietnam or Yemen or Yugoslavia or Zambia or Zimbabwe or Southern African Development Community or East African Community or West African Health Organisation or "resource-poor settings" or "resource-limited settings" or "low-resource settings" or "tropic*").mp. |
| 7 | 1 and 2 and 3 and 4 and 5 and 6 |
| 8 | 1 and 2 and 3 and 4 and 6 |
| 9 | limit 7 to yr="2012-current" |
| 10 | limit 8 to yr="2012-current" |

| **Embase Classic+Embase <1947 to 2022 July 31>** | |
| --- | --- |
|  |  |
| 1 | (postpartum or post-partum or postnatal or post-natal or puerperium or "after deliver*" or "after childbirth" or "after birth*" or "since birth*" or "following deliver*" or "following childbirth" or "following birth*").mp. or exp Postpartum Period/ [mp=title, abstract, heading word, drug trade name, original title, device manufacturer, drug manufacturer, device trade name, keyword heading word, floating subheading word, candidate term word] |
| 2 | (contracept* or "family planning" or "birth control" or "fertility control" or "birth regulation" or "fertility regulation" or (birth* adj2 spac*) or (child adj2 spac*) or (childbirth adj2 spac*) or (birth* adj2 timi*) or (pregnanc* adj2 timi*) or (pregnanc* adj2 spac*) or (childbirth adj3 interval*) or (birth* adj3 interval*) or (pregnanc* adj3 interval*) or (conce* adj2 interval*)).mp. or exp Contraception/ or exp Contraceptive Devices/ or depo? Medroxyprogesterone.mp. or Depo-Provera.mp. or Sayana Press.mp. or IUD.mp. or IUCD.mp. or IUS.mp. or intra?uterine device*.mp. or intra?uterine system*.mp. or oral contraceptive pill*.mp. or hormonal contraceptive pill*.mp. or birth control pill.mp. or mini?pill.mp. or progesterone?only pill.mp. or emergency contraceptive.mp. or cervical cap.mp. or cervical caps.mp. or vaginal diaphragm*.mp. or vaginal ring*.mp. or implant*.mp. or subdermal implant*.mp. or implanon.mp. or jadelle.mp. or norplant*.mp. or sino?implant.mp. or sterili?ation.mp. or vasectomy.mp. or contraception behavio?r.mp. or long-acting reversible contraception.mp. or LARCS.mp. or family planning/ or birth control/ or contraception/ or Contraception Behavio?r/ or Family Planning Services/ [mp=title, abstract, heading word, drug trade name, original title, device manufacturer, drug manufacturer, device trade name, keyword heading word, floating subheading word, candidate term word] |
| 3 | (method* or interven* or meet* or consult* or advi* or counsel* or visit* or session* or educat* or communicat* or involve* or communit* or facilit* or service* or deliver* or provi* or program* or campaign* or scheme* or outreach*).mp. or exp intervention studies/ or (Family planning counsel?ing or family planning information or family planning advice or contraceptive counsel?ing).mp. [mp=title, abstract, heading word, drug trade name, original title, device manufacturer, drug manufacturer, device trade name, keyword heading word, floating subheading word, candidate term word] |
| 4 | (ANC or PNC or ((antenatal or prenatal or pre?natal or pregnancy) and care)).mp. or exp Prenatal care/ [mp=title, abstract, heading word, drug trade name, original title, device manufacturer, drug manufacturer, device trade name, keyword heading word, floating subheading word, candidate term word] |
| 5 | (trial* or control* or randomi* or evaluat* or prospective or longitudinal or blind* or single-blind* or "single blind*" or double-blind* or "double blind*" or "before and after" or crossover or cross-over or cluster* or placebo or arm or arms or "quasi?experiment*" or "pre?post" or meta-analysis or synthesis or literature or published or extraction or search or review*).mp. or (quasiexperiment$2 or quasi experiment$2).ti,ab. or exogenous variation$1.ti,ab. or natural experiment$2.ti,ab. or Matched controls.ti,ab. or Counterfactual outcome$1.ti,ab. or Rubin causal model$1.ti,ab. or potential outcomes model$1.ti,ab. or (Identification adj (strategy or assumptions or conditions)).ti,ab. or (conditional adj (independence or ignorability)).ti,ab. or unobserved heterogeneity.ti,ab. or Unconfoundness.ti,ab. or Confounding.ti,ab. or (instrumental variable$1 adj (analysis or analyses or estimation)).ti,ab. or (overidentification or overidentifying).ti,ab. or regression discontinuity analys$2.ti,ab. or ((balancing or imbalance or balanced or imbalanced) adj3 covariates).ti,ab. or interrupted time series.ti,ab. or difference studies.ti,ab. or (controlled adj3 before adj3 after).ti,ab. or ((exact or score or genetic or nearest neighbor or nearest neighbour or caliper or radius or kernel density or blocking or stratification of interval) adj3 matching).ti,ab. or (Inverse probability weight$ adj4 estimat$).ti,ab. or (doubly robust adj4 (regression or estimate$)).ti,ab. or ((treatment or switching or selection or selectivity) adj3 regression).ti,ab. or (selection model or selectivity model).ti,ab. or (heckit model or heckman sample selection).ti,ab. or selection correction.ti,ab. or two stage residual inclusion.ti,ab. or regression discontinuity.ti,ab. or (sharp design or fuzzy design).ti,ab. or Forcing variable$1.ti,ab. or (difference$1 adj3 difference$1).ti,ab. or (change$1 adj3 change$1).ti,ab. or (Fixed effects and panel data).ti,ab. or full information maximum likelihood.ti,ab. or ((health or economic) adj shock$1).ti,ab. or natural controls.ti,ab. [mp=title, abstract, heading word, drug trade name, original title, device manufacturer, drug manufacturer, device trade name, keyword heading word, floating subheading word, candidate term word] |
| 6 | (emerging country or emerging countries or emerging nation or emerging nations or emerging population or emerging populations developing country or developing countries or developing nation or developing nations or developing population or developing populations or developing world or less developed country or less developed countries or less developed nation or less developed nations or less developed population or less developed populations or less developed world or lesser developed country or lesser developed countries or lesser developed nation or lesser developed nations or lesser developed population or lesser developed populations or lesser developed world or under developed country or under developed countries or under developed nation or under developed nations or under developed population or under developed populations or under developed world or underdeveloped country or underdeveloped countries or underdeveloped nation or underdeveloped nations or underdeveloped population or underdeveloped populations or underdeveloped world or middle income country or middle income countries or middle income nation or middle income nations or middle income population or middle income populations or low income country or low income countries or low income nation or low income nations or low income population or low income populations or lower income country or lower income countries or lower income nation or lower income nations or lower income population or lower income populations or underserved country or underserved countries or underserved nation or underserved nations or underserved population or underserved populations or underserved world or under served country or under served countries or under served nation or under served nations or under served population or under served populations or under served world or deprived country or deprived countries or deprived nation or deprived nations or deprived population or deprived populations or deprived world or poor country or poor countries or poor nation or poor nations or poor population or poor populations or poor world or poorer country or poorer countries or poorer nation or poorer nations or poorer population or poorer populations or poorer world or developing economy or developing economies or less developed economy or less developed economies or lesser developed economy or lesser developed economies or under developed economy or under developed economies or underdeveloped economy or underdeveloped economies or middle income economy or middle income economies or low income economy or low income economies or lower income economy or lower income economies or low gdp or low gnp or low gross domestic or low gross national or lower gdp or lower gnp or lower gross domestic or lower gross national or lmic or lmics or third world or lami country or lami countries or transitional country or transitional countries or Africa or Asia or Caribbean or West Indies or South America or Latin America or Central America or Atlantic Islands or Pacific Islands or Indian Ocean Islands or Eastern Europe or Afghanistan or Albania or Algeria or Angola or Antigua or Barbuda or Argentina or Armenia or Armenian or Aruba or Azerbaijan or Bahrain or Bangladesh or Barbados or Benin or Byelarus or Byelorussian or Belarus or Belorussian or Belorussia or Belize or Bhutan or Bolivia or Bosnia or Herzegovina or Hercegovina or Botswana or Brasil or Brazil or Bulgaria or Burkina Faso or Burkina Fasso or Upper Volta or Burundi or Urundi or Cambodia or Khmer Republic or Kampuchea or Cameroon or Cameroons or Cameron or Camerons or Cape Verde or Central African Republic or Chad or Chile or China or Colombia or Comoros or Comoro Islands or Comores or Mayotte or Congo or Zaire or Costa Rica or Cote d'Ivoire or Ivory Coast or Croatia or Cuba or Cyprus or Czechoslovakia or Czech Republic or Slovakia or Slovak Republic or Djibouti or French Somaliland or Dominica or Dominican Republic or East Timor or East Timur or Timor Leste or Ecuador or Egypt or United Arab Republic or El Salvador or Eritrea or Estonia or Ethiopia or Fiji or Gabon or Gabonese Republic or Gambia or Gaza or Georgia Republic or Georgian Republic or Ghana or Gold Coast or Greece or Grenada or Guatemala or Guinea or Guam or Guiana or Guyana or Haiti or Honduras or Hungary or India or Maldives or Indonesia or Iran or Iraq or Jamaica or Jordan or Kazakhstan or Kazakh or Kenya or Kiribati or Korea or Kosovo or Kyrgyzstan or Kirghizia or Kyrgyz Republic or Kirghiz or Kirgizstan or Lao PDR or Laos or Latvia or Lebanon or Lesotho or Basutoland or Liberia or Libya or Lithuania or Macedonia or Madagascar or Malagasy Republic or Malaysia or Malaya or Malay or Sabah or Sarawak or Malawi or Nyasaland or Mali or Malta or Marshall Islands or Mauritania or Mauritius or Agalega Islands or Melanesia or Mexico or Micronesia or Middle East or Moldova or Moldovia or Moldovian or Mongolia or Montenegro or Morocco or Ifni or Mozambique or Myanmar or Myanma or Burma or Namibia or Nepal or Netherlands Antilles or New Caledonia or Nicaragua or Niger or Nigeria or Northern Mariana Islands or Oman or Muscat or Pakistan or Palau or Palestine or Panama or Paraguay or Peru or Philippines or Philipines or Phillipines or Phillippines or Poland or Portugal or Puerto Rico or Romania or Rumania or Roumania or Russia or Russian or Rwanda or Ruanda or Saint Kitts or St Kitts or Nevis or Saint Lucia or St Lucia or Saint Vincent or St Vincent or Grenadines or Samoa or Samoan Islands or Navigator Island or Navigator Islands or Sao Tome or Saudi Arabia or Senegal or Serbia or Montenegro or Seychelles or Sierra Leone or Slovenia or Sri Lanka or Ceylon or Solomon Islands or Somalia or Sudan or Suriname or Surinam or Swaziland or Syria or Syrian or Tajikistan or Tadzhikistan or Tadjikistan or Tadzhik or Tanzania or Thailand or Togo or Togolese Republic or Tonga or Trinidad or Tobago or Tunisia or Turkey or Turkmenistan or Turkmen or Tuvalu or Uganda or Ukraine or Uruguay or USSR or Soviet Union or Soviet Socialist Republics or Uzbekistan or Uzbek or Vanuatu or New Hebrides or Venezuela or Vietnam or Viet Nam or West Bank or Yemen or Yugoslavia or Zambia or Zimbabwe or Rhodesia or Developing Countries or Africa or Asia or Caribbean Region or West Indies or South America or Latin America or Central America or Atlantic Islands or Pacific Islands or Indian Ocean Islands or Afghanistan or Albania or Algeria or American Samoa or Angola or "Antigua and Barbuda" or Argentina or Armenia or Azerbaijan or Bahrain or Baltic States or Bangladesh or Barbados or Benin or Belarus or Belize or Bhutan or Bolivia or Bosnia-Herzegovina or Botswana or Brazil or Bulgaria or Burkina Faso or Burundi or Cambodia or Cameroon or Cape Verde or Central African Republic or Chad or Chile or China or Colombia or Comoros or Congo or Costa Rica or Cote d'Ivoire or Croatia or Cuba or Cyprus or Czechoslovakia or Czech Republic or Slovakia or Djibouti or Congo or Korea or Dominica or Dominican Republic or East Timor or Ecuador or Egypt or El Salvador or Eritrea or Estonia or Ethiopia or Equatorial Guinea or Fiji or French Guiana or Gabon or Gambia or Georgia or Ghana or Greece or Grenada or Guatemala or Guinea or Guinea-Bissau or Guam or Guyana or Haiti or Honduras or Hungary or Samoa or India or Indonesia or Iran or Iraq or Jamaica or Jordan or Kazakhstan or Kenya or Korea or Kyrgyzstan or Laos or Latvia or Lebanon or Lesotho or Liberia or Libya or Lithuania or Macedonia or Madagascar or Malawi or Malaysia or Mali or Malta or Mauritania or Mauritius or Melanesia or Mexico or Micronesia or Middle East or Moldova or Mongolia or Montenegro or Morocco or Mozambique or Myanmar or Namibia or Nepal or Netherlands Antilles or New Caledonia or Nicaragua or Niger or Nigeria or Oman or Pakistan or Palau or Panama or Papua New Guinea or Paraguay or Peru or Philippines or Poland or Portugal or Puerto Rico or Romania or Russia or Russia or Rwanda or "Saint Kitts and Nevis" or Saint Lucia or Grenadines or Samoa or Saudi Arabia or Senegal or Serbia or Montenegro or Seychelles or Sierra Leone or Slovenia or Sri Lanka or Somalia or South Africa or Sudan or Suriname or Swaziland or Syria or Tajikistan or Tanzania or Thailand or Togo or Tonga or "Trinidad and Tobago" or Tunisia or Turkey or Turkmenistan or Uganda or Ukraine or Uruguay or USSR or Uzbekistan or Vanuatu or Venezuela or Vietnam or Yemen or Yugoslavia or Zambia or Zimbabwe or Southern African Development Community or East African Community or West African Health Organisation or "resource-poor settings" or "resource-limited settings" or "low-resource settings" or "tropic*").mp. |
| 7 | 1 and 2 and 3 and 4 and 5 and 6 |
| 8 | 1 and 2 and 3 and 4 and 6 |
| 9 | limit 7 to yr="2012-current" |
| 10 | limit 8 to yr="2012-current" |

| **Global Health <1910 to 2022 Week 30>** | |
| --- | --- |
|  |  |
| 1 | (postpartum or post-partum or postnatal or post-natal or puerperium or "after deliver*" or "after childbirth" or "after birth*" or "since birth*" or "following deliver*" or "following childbirth" or "following birth*").mp. or exp Postpartum Period/ [mp=abstract, title, original title, heading words, cabicodes words] |
| 2 | (contracept* or "family planning" or "birth control" or "fertility control" or "birth regulation" or "fertility regulation" or (birth* adj2 spac*) or (child adj2 spac*) or (childbirth adj2 spac*) or (birth* adj2 timi*) or (pregnanc* adj2 timi*) or (pregnanc* adj2 spac*) or (childbirth adj3 interval*) or (birth* adj3 interval*) or (pregnanc* adj3 interval*) or (conce* adj2 interval*)).mp. or exp Contraception/ or exp Contraceptive Devices/ or depo? Medroxyprogesterone.mp. or Depo-Provera.mp. or Sayana Press.mp. or IUD.mp. or IUCD.mp. or IUS.mp. or intra?uterine device*.mp. or intra?uterine system*.mp. or oral contraceptive pill*.mp. or hormonal contraceptive pill*.mp. or birth control pill.mp. or mini?pill.mp. or progesterone?only pill.mp. or emergency contraceptive.mp. or cervical cap.mp. or cervical caps.mp. or vaginal diaphragm*.mp. or vaginal ring*.mp. or implant*.mp. or subdermal implant*.mp. or implanon.mp. or jadelle.mp. or norplant*.mp. or sino?implant.mp. or sterili?ation.mp. or vasectomy.mp. or contraception behavio?r.mp. or long-acting reversible contraception.mp. or LARCS.mp. or family planning/ or birth control/ or contraception/ or Contraception Behavio?r/ or Family Planning Services/ [mp=abstract, title, original title, heading words, cabicodes words] |
| 3 | (method* or interven* or meet* or consult* or advi* or counsel* or visit* or session* or educat* or communicat* or involve* or communit* or facilit* or service* or deliver* or provi* or program* or campaign* or scheme* or outreach*).mp. or exp intervention studies/ or (Family planning counsel?ing or family planning information or family planning advice or contraceptive counsel?ing).mp. [mp=abstract, title, original title, heading words, cabicodes words] |
| 4 | (ANC or PNC or ((antenatal or prenatal or pre?natal or pregnancy) and care)).mp. or exp Prenatal care/ [mp=abstract, title, original title, heading words, cabicodes words] |
| 5 | (trial* or control* or randomi* or evaluat* or prospective or longitudinal or blind* or single-blind* or "single blind*" or double-blind* or "double blind*" or "before and after" or crossover or cross-over or cluster* or placebo or arm or arms or "quasi?experiment*" or "pre?post" or meta-analysis or synthesis or literature or published or extraction or search or review*).mp. or (quasiexperiment$2 or quasi experiment$2).ti,ab. or exogenous variation$1.ti,ab. or natural experiment$2.ti,ab. or Matched controls.ti,ab. or Counterfactual outcome$1.ti,ab. or Rubin causal model$1.ti,ab. or potential outcomes model$1.ti,ab. or (Identification adj (strategy or assumptions or conditions)).ti,ab. or (conditional adj (independence or ignorability)).ti,ab. or unobserved heterogeneity.ti,ab. or Unconfoundness.ti,ab. or Confounding.ti,ab. or (instrumental variable$1 adj (analysis or analyses or estimation)).ti,ab. or (overidentification or overidentifying).ti,ab. or regression discontinuity analys$2.ti,ab. or ((balancing or imbalance or balanced or imbalanced) adj3 covariates).ti,ab. or interrupted time series.ti,ab. or difference studies.ti,ab. or (controlled adj3 before adj3 after).ti,ab. or ((exact or score or genetic or nearest neighbor or nearest neighbour or caliper or radius or kernel density or blocking or stratification of interval) adj3 matching).ti,ab. or (Inverse probability weight$ adj4 estimat$).ti,ab. or (doubly robust adj4 (regression or estimate$)).ti,ab. or ((treatment or switching or selection or selectivity) adj3 regression).ti,ab. or (selection model or selectivity model).ti,ab. or (heckit model or heckman sample selection).ti,ab. or selection correction.ti,ab. or two stage residual inclusion.ti,ab. or regression discontinuity.ti,ab. or (sharp design or fuzzy design).ti,ab. or Forcing variable$1.ti,ab. or (difference$1 adj3 difference$1).ti,ab. or (change$1 adj3 change$1).ti,ab. or (Fixed effects and panel data).ti,ab. or full information maximum likelihood.ti,ab. or ((health or economic) adj shock$1).ti,ab. or natural controls.ti,ab. [mp=abstract, title, original title, heading words, cabicodes words] |
| 6 | (emerging country or emerging countries or emerging nation or emerging nations or emerging population or emerging populations developing country or developing countries or developing nation or developing nations or developing population or developing populations or developing world or less developed country or less developed countries or less developed nation or less developed nations or less developed population or less developed populations or less developed world or lesser developed country or lesser developed countries or lesser developed nation or lesser developed nations or lesser developed population or lesser developed populations or lesser developed world or under developed country or under developed countries or under developed nation or under developed nations or under developed population or under developed populations or under developed world or underdeveloped country or underdeveloped countries or underdeveloped nation or underdeveloped nations or underdeveloped population or underdeveloped populations or underdeveloped world or middle income country or middle income countries or middle income nation or middle income nations or middle income population or middle income populations or low income country or low income countries or low income nation or low income nations or low income population or low income populations or lower income country or lower income countries or lower income nation or lower income nations or lower income population or lower income populations or underserved country or underserved countries or underserved nation or underserved nations or underserved population or underserved populations or underserved world or under served country or under served countries or under served nation or under served nations or under served population or under served populations or under served world or deprived country or deprived countries or deprived nation or deprived nations or deprived population or deprived populations or deprived world or poor country or poor countries or poor nation or poor nations or poor population or poor populations or poor world or poorer country or poorer countries or poorer nation or poorer nations or poorer population or poorer populations or poorer world or developing economy or developing economies or less developed economy or less developed economies or lesser developed economy or lesser developed economies or under developed economy or under developed economies or underdeveloped economy or underdeveloped economies or middle income economy or middle income economies or low income economy or low income economies or lower income economy or lower income economies or low gdp or low gnp or low gross domestic or low gross national or lower gdp or lower gnp or lower gross domestic or lower gross national or lmic or lmics or third world or lami country or lami countries or transitional country or transitional countries or Africa or Asia or Caribbean or West Indies or South America or Latin America or Central America or Atlantic Islands or Pacific Islands or Indian Ocean Islands or Eastern Europe or Afghanistan or Albania or Algeria or Angola or Antigua or Barbuda or Argentina or Armenia or Armenian or Aruba or Azerbaijan or Bahrain or Bangladesh or Barbados or Benin or Byelarus or Byelorussian or Belarus or Belorussian or Belorussia or Belize or Bhutan or Bolivia or Bosnia or Herzegovina or Hercegovina or Botswana or Brasil or Brazil or Bulgaria or Burkina Faso or Burkina Fasso or Upper Volta or Burundi or Urundi or Cambodia or Khmer Republic or Kampuchea or Cameroon or Cameroons or Cameron or Camerons or Cape Verde or Central African Republic or Chad or Chile or China or Colombia or Comoros or Comoro Islands or Comores or Mayotte or Congo or Zaire or Costa Rica or Cote d'Ivoire or Ivory Coast or Croatia or Cuba or Cyprus or Czechoslovakia or Czech Republic or Slovakia or Slovak Republic or Djibouti or French Somaliland or Dominica or Dominican Republic or East Timor or East Timur or Timor Leste or Ecuador or Egypt or United Arab Republic or El Salvador or Eritrea or Estonia or Ethiopia or Fiji or Gabon or Gabonese Republic or Gambia or Gaza or Georgia Republic or Georgian Republic or Ghana or Gold Coast or Greece or Grenada or Guatemala or Guinea or Guam or Guiana or Guyana or Haiti or Honduras or Hungary or India or Maldives or Indonesia or Iran or Iraq or Jamaica or Jordan or Kazakhstan or Kazakh or Kenya or Kiribati or Korea or Kosovo or Kyrgyzstan or Kirghizia or Kyrgyz Republic or Kirghiz or Kirgizstan or Lao PDR or Laos or Latvia or Lebanon or Lesotho or Basutoland or Liberia or Libya or Lithuania or Macedonia or Madagascar or Malagasy Republic or Malaysia or Malaya or Malay or Sabah or Sarawak or Malawi or Nyasaland or Mali or Malta or Marshall Islands or Mauritania or Mauritius or Agalega Islands or Melanesia or Mexico or Micronesia or Middle East or Moldova or Moldovia or Moldovian or Mongolia or Montenegro or Morocco or Ifni or Mozambique or Myanmar or Myanma or Burma or Namibia or Nepal or Netherlands Antilles or New Caledonia or Nicaragua or Niger or Nigeria or Northern Mariana Islands or Oman or Muscat or Pakistan or Palau or Palestine or Panama or Paraguay or Peru or Philippines or Philipines or Phillipines or Phillippines or Poland or Portugal or Puerto Rico or Romania or Rumania or Roumania or Russia or Russian or Rwanda or Ruanda or Saint Kitts or St Kitts or Nevis or Saint Lucia or St Lucia or Saint Vincent or St Vincent or Grenadines or Samoa or Samoan Islands or Navigator Island or Navigator Islands or Sao Tome or Saudi Arabia or Senegal or Serbia or Montenegro or Seychelles or Sierra Leone or Slovenia or Sri Lanka or Ceylon or Solomon Islands or Somalia or Sudan or Suriname or Surinam or Swaziland or Syria or Syrian or Tajikistan or Tadzhikistan or Tadjikistan or Tadzhik or Tanzania or Thailand or Togo or Togolese Republic or Tonga or Trinidad or Tobago or Tunisia or Turkey or Turkmenistan or Turkmen or Tuvalu or Uganda or Ukraine or Uruguay or USSR or Soviet Union or Soviet Socialist Republics or Uzbekistan or Uzbek or Vanuatu or New Hebrides or Venezuela or Vietnam or Viet Nam or West Bank or Yemen or Yugoslavia or Zambia or Zimbabwe or Rhodesia or Developing Countries or Africa or Asia or Caribbean Region or West Indies or South America or Latin America or Central America or Atlantic Islands or Pacific Islands or Indian Ocean Islands or Afghanistan or Albania or Algeria or American Samoa or Angola or "Antigua and Barbuda" or Argentina or Armenia or Azerbaijan or Bahrain or Baltic States or Bangladesh or Barbados or Benin or Belarus or Belize or Bhutan or Bolivia or Bosnia-Herzegovina or Botswana or Brazil or Bulgaria or Burkina Faso or Burundi or Cambodia or Cameroon or Cape Verde or Central African Republic or Chad or Chile or China or Colombia or Comoros or Congo or Costa Rica or Cote d'Ivoire or Croatia or Cuba or Cyprus or Czechoslovakia or Czech Republic or Slovakia or Djibouti or Congo or Korea or Dominica or Dominican Republic or East Timor or Ecuador or Egypt or El Salvador or Eritrea or Estonia or Ethiopia or Equatorial Guinea or Fiji or French Guiana or Gabon or Gambia or Georgia or Ghana or Greece or Grenada or Guatemala or Guinea or Guinea-Bissau or Guam or Guyana or Haiti or Honduras or Hungary or Samoa or India or Indonesia or Iran or Iraq or Jamaica or Jordan or Kazakhstan or Kenya or Korea or Kyrgyzstan or Laos or Latvia or Lebanon or Lesotho or Liberia or Libya or Lithuania or Macedonia or Madagascar or Malawi or Malaysia or Mali or Malta or Mauritania or Mauritius or Melanesia or Mexico or Micronesia or Middle East or Moldova or Mongolia or Montenegro or Morocco or Mozambique or Myanmar or Namibia or Nepal or Netherlands Antilles or New Caledonia or Nicaragua or Niger or Nigeria or Oman or Pakistan or Palau or Panama or Papua New Guinea or Paraguay or Peru or Philippines or Poland or Portugal or Puerto Rico or Romania or Russia or Russia or Rwanda or "Saint Kitts and Nevis" or Saint Lucia or Grenadines or Samoa or Saudi Arabia or Senegal or Serbia or Montenegro or Seychelles or Sierra Leone or Slovenia or Sri Lanka or Somalia or South Africa or Sudan or Suriname or Swaziland or Syria or Tajikistan or Tanzania or Thailand or Togo or Tonga or "Trinidad and Tobago" or Tunisia or Turkey or Turkmenistan or Uganda or Ukraine or Uruguay or USSR or Uzbekistan or Vanuatu or Venezuela or Vietnam or Yemen or Yugoslavia or Zambia or Zimbabwe or Southern African Development Community or East African Community or West African Health Organisation or "resource-poor settings" or "resource-limited settings" or "low-resource settings" or "tropic*").mp. |
| 7 | 1 and 2 and 3 and 4 and 5 and 6 |
| 8 | 1 and 2 and 3 and 4 and 6 |
| 9 | limit 7 to yr="2012-current" |
| 10 | limit 8 to yr="2012-current" |
